# Impact of industry sponsorship on the quality of systematic reviews of vaccines: a cross-sectional analysis of studies published from 2016 to 2019

**DOI:** 10.1186/s13643-022-02051-x

**Published:** 2022-08-22

**Authors:** Dawid Pieper, Irma Hellbrecht, Linlu Zhao, Clemens Baur, Georgia Pick, Sarah Schneider, Thomas Harder, Kelsey Young, Andrea C. Tricco, Ella Westhaver, Matthew Tunis

**Affiliations:** 1 Institute for Research in Operative Medicine, Evidence-Based Health Services Research, Faculty of Health, School of Medicine, Witten/Herdecke University, Ostmerheimer Str. 200, building 38, 51109 Cologne, Germany; 2 Faculty of Health Sciences Brandenburg, Brandenburg Medical School (Theodor Fontane), Institute for Health Services and Health System Research, Rüdersdorf, Germany; 3 Center for Health Services Research, Brandenburg Medical School (Theodor Fontane), Rüdersdorf, Germany; 4 Institute of Health Economics and Clinical Epidemiology, University of Cologne, Cologne, Germany; 5 Health Canada and Public Health Agency of Canada, Ottawa, Ontario Canada; 6 Robert Koch Institute, Berlin, Germany; 7 Li Ka Shing Knowledge Institute, St Michael’s Hospital, Unity Health Toronto, Toronto, Ontario Canada; 8 Epidemiology Division of the Dalla Lana School of Public Health and the Institute for Health, University of Toronto, Toronto, Ontario Canada; 9 Policy, Management, and Evaluation, University of Toronto, Toronto, Ontario Canada; 10 Queen’s Collaboration for Health Care Quality Joanna Briggs Institute Centre of Excellence, School of Nursing, Queen’s University, Kingsto, Ontario Canada

**Keywords:** Systematic Reviews, Meta-analysis, Methodological Quality, AMSTAR 2, Funding sources

## Abstract

**Background:**

Systematic reviews (SRs) provide the highest level of evidence and inform evidence-based decision making in health care. Earlier studies found association with industry to be negatively associated with methodological quality of SRs. However, this has not been investigated in SRs on vaccines.

**Methods:**

We performed a systematic literature search using MEDLINE and EMBASE in March 2020. The results were restricted to those published between 2016 and 2019 with no language restrictions. Study characteristics were extracted by one person and checked by an experienced reviewer. The methodological quality of the SRs was assessed with the AMSTAR 2 tool by multiple reviewers after a calibration exercise was performed. A summary score for each SR was calculated. The Mann-Whitney *U* test and Fisher’s exact test were performed to compare both groups.

**Results:**

Out of 185 SRs that met all inclusion criteria, 27 SRs were industry funded. Those were matched with 30 non-industry funded SRs resulting in a total sample size of 57. The mean AMSTAR 2 summary score across all SRs was 0.49. Overall, the median AMSTAR 2 summary score was higher for the non-industry funded SRs than for the industry-funded SRs (0.62 vs. 0.36; *p* < .00001). Lower ratings for industry funded SRs were consistent across all but one AMSTAR 2 item, though significantly lower only for three specific items.

**Conclusion:**

The methodological quality of SRs in vaccination is comparable to SRs in other fields, while it is still suboptimal. We are not able to provide a satisfactory explanation why industry funded SRs had a lower methodological quality than non-industry funded SRs over recent years. Industry funding is an important indicator of methodological quality for vaccine SRs and should be carefully considered when appraising SR quality.

**Supplementary Information:**

The online version contains supplementary material available at 10.1186/s13643-022-02051-x.

## Introduction

Systematic reviews (SRs) provide the highest level of evidence and inform evidence-based decision making in health care. The number of SRs in the field of vaccines increased steadily over the last decade [1]. In contrast to other topics, SRs in the field of vaccination come with several unique methodological challenges. In particular, this includes the consideration of non-randomized studies, differentiation between outcomes such as immunogenicity and efficacy or effectiveness, safety outcomes, and age-specific effects [2–6].

According to the World Health Organization (WHO), National Immunization Technical Advisory Groups (NITAGs) should apply evidence-based approaches in the process of developing vaccination recommendations [7, 8]. Given their central importance in this process, SRs with methodological flaws might bias decisions and negatively affect population health. Research has repeatedly shown that the methodological quality of SRs is generally suboptimal, while too many SRs are published on the same topics at the same time [9]. This makes the use of evidence syntheses such as SRs much more complicated [1]. Methodological quality assessment tools can facilitate choosing the most suitable SR for decision-making, although contextual factors such as PICOS (population, intervention, comparator, outcome, and setting) alignment can also play a role.

The methodological quality of SRs can be associated with several aspects. For example, aspects positively associated with the methodological quality were found to be prospective registration of the SRs [10–12], performing meta-analysis [11, 13], including randomized controlled trials in the SRs [13], SRs that were Cochrane reviews [13], and journal impact factor [14] across different fields. Funding can also play a role: a recent Cochrane review found that SRs on drugs or devices with financial conflicts of interest more often have favorable conclusions and tend to have lower methodological quality than SRs without financial conflicts of interest [15]. Earlier studies found association with industry to be negatively associated with methodological quality of SRs [16, 17]. However, they did not consider SRs on vaccines.

The aim of this study was to compare SRs of vaccine intervention studies that were sponsored by industry, either through authorship, funding, or a combination thereof, compared to non-industry sources to evaluate whether industry sponsorship influences the methodological quality of SRs of vaccines. Understanding the relationship between funding and quality can help NITAGs to choose among available SRs to support the development of vaccination recommendations. This might also be important during the ongoing SARS-CoV-2 pandemic since vaccine studies are quickly multiplying and soon SRs will emerge, including from industry, to inform program policies.

## Methods

This is a cross-sectional study using a matched design. There was an unpublished a priori protocol for this study (see Additional file 1).

### Eligibility criteria

To meet eligibility criteria, SRs had to search bibliographic databases and identify and select relevant studies based on specified eligibility criteria. To be eligible, a SR had to investigate at least one of the following outcomes: (1) efficacy, effectiveness; (2) safety; or (3) immunogenicity of vaccines. All populations and settings were considered. The intervention of interest was vaccination in humans with one or more specific vaccines or with a class of vaccines (e.g., vaccines against pneumococcal disease). Therapeutic vaccines and passive immunization agents were not considered. Empty SRs (i.e., SRs having no eligible inclusions) were excluded as well as clinical practice guidelines, health technology assessment reports, and other types of reviews (e.g., narrative, scoping) together with burden of disease studies, economic assessments of vaccination, risk assessments of vaccination, vaccine-related modeling, qualitative assessments of vaccination and vaccine program evaluation even if they performed literature searches. In addition, we only considered SRs published between 2016 and 2019 to focus on current trends and standards.

### Information sources and search strategy

We performed a systematic literature search using MEDLINE and EMBASE on the OVID platform. The search strategy was developed in collaboration with a librarian from the Health Library of Health Canada and the Public Health Agency of Canada (EW) and was peer reviewed by a second librarian using the PRESS statement [18]. The search combined vaccine and immunization keywords and subject headings with an adaption of the CADTH systematic review filter [19]. The results were restricted to those published between 2016 and 2019 with no language restrictions. The search was performed on March 30, 2020. The complete search strategy can be found in Additional file 2.

### Study selection

References were screened by title and abstract first. The full text of articles deemed relevant was retrieved and assessed for eligibility. Piloting of the study selection process took place for both title/abstract and full-text screening. At each step screening was performed by one reviewer (CB, GP, IH, SaS) and verified by a second reviewer (DP). Differences were discussed until consensus was reached or a third reviewer was brought in to mediate. SRs were screened by year, i.e., each first reviewer (CB, GP, IH, SaS) examined only 1 year.

SRs matching was applied, as the number of industry-funded SRs was expected to be smaller than the number of non-industry funded SRs. We determined funding status by checking the published funding statements and conflicts of interest statements. For each industry-funded SR (case) a control was randomly drawn (using the RAND function in Excel) from non-industry funded SRs published in the same year. No other matching variables were applied. We planned to apply 1:1 matching. However, 1:2 matching was applied in the 2016 subset due a low number of cases.

### Data extraction

After piloting of data extraction sheets, each reviewer (CB, GP, IH, SaS) abstracted data from eligible studies into an Excel extraction form and data were checked by a second reviewer (DP). Again, differences were discussed until consensus was reached or through third reviewer mediation. We abstracted data for general characteristics, impact factor (2019), included studies, searched databases, quality appraisal tool, and evidence synthesis.

### Quality assessment

All SRs were assessed with the AMSTAR 2 tool [20]. AMSTAR 2 builds up on the former used AMSTAR [21] and has been designed to assess the methodological quality of SRs. AMSTAR 2 consists of 16 items allowing for the answer “yes” or “no” and “partial yes” for some items. AMSTAR 2 allows for rating the overall confidence in the results and was found to be valid and moderately reliable [22–25]. A calibration exercise among all reviewers (CB, GP, IH, SaS) and a senior author (DP) experienced with SRs and AMSTAR 2 was performed on 4 SRs not meeting eligibility criteria to ensure consistency. In addition, an internal guidance document was developed, approved, and used throughout the process. A single reviewer (CB, GP, IH, SaS) assessed the methodological quality of the included SRs. Where needed, reviewers reached out to the senior author in case of any uncertainty where no guidance was available either from the AMSTAR 2 tool guidance document or the internal guidance document. Those who were assessing the study quality were not blinded to the industry status of each study, due to logistical constraints.

### Data analysis

We did not assess the overall confidence using AMSTAR 2 due to potential floor effects [26–28] and to allow for item-level analyses but did rely on summary scores. We calculated a summary score for each SR. Items from the AMSTAR 2 tool scoring “yes” obtained 1 point, whereas items scoring “partial yes” obtained 0.5 points. Due to the fact that the number of relevant items can vary (e.g., items on meta-analysis can only be judged if meta-analysis was performed) we divided the summed points by the highest achievable points per SR.

We used the Mann-Whitney *U* test to compare summary scores between both groups. At item level, we have replaced the originally planned chi-squared test by Fisher’s exact test due to low cell numbers for some items. For all item-level analyses, we combined “yes” and “partial yes” into one category and in this case considered the item to be completely fulfilled. *P*-values < 0.05 were considered to be statistically significant.

## Results

### Study selection

**Fig. 1:**
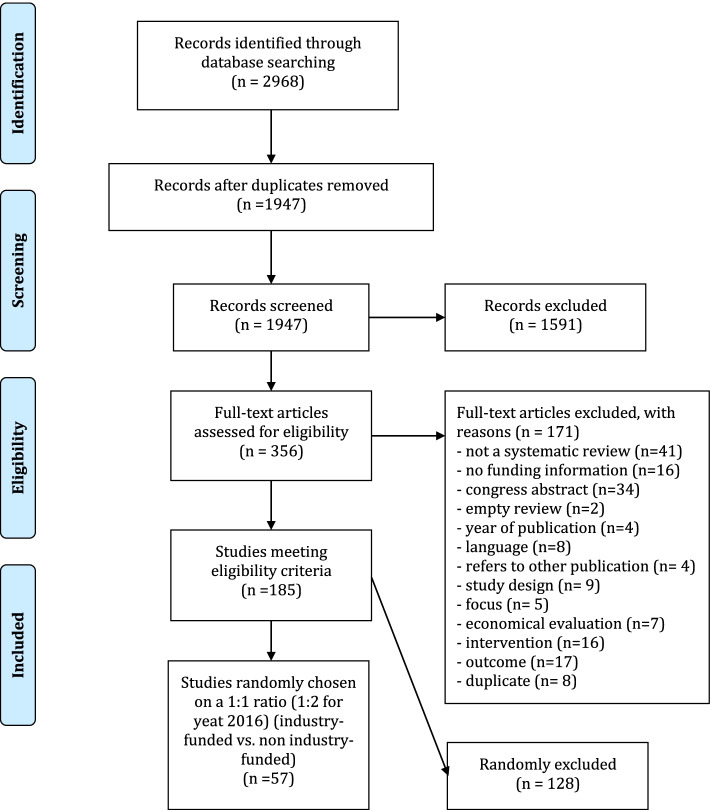
Flow chart

The systematic literature search of the electronic databases identified 2968 references. After screening titles and abstracts, we retrieved full-text of 356 articles. Of these, 185 SRs met all inclusion criteria. Only 27 (15%) SRs were industry funded [29–55]. After matching, 30 non-industry funded SRs [56–85] were randomly chosen resulting in a total sample size of 57 (see Fig. 1). The remaining 128 SRs were not considered in the analysis. A list of excluded references can be found in Additional file 3.

### Study characteristics

The selected 57 SRs comprised a total of 1294 studies (without considering overlap of studies between SRs). The median number of included studies was 15.0 (range 3–150), with industry funded SR including more studies (median 24 vs. 12.5). The median journal impact factor (JIF) was 3.14 (range 1.18–24.45; *n* = 54, three articles were published in a journal without an impact factor). Median JIF was slightly higher in industry funded SRs (3.656 vs. 3). The median number of databases searched was 4 in both groups. The majority of SRs included non-randomized studies (44/57, 77%). Network meta-analysis was conducted in one study only [49]. The SR interventions represented a broad range of vaccine-preventable infectious diseases dominated by influenza (14/57, 25%), *Streptococcus pneumoniae* (12/57, 21%), and human papillomavirus infection (8/57, 14%) and also including hepatitis, tetanus, diphtheria, pertussis, poliomyelitis, rotavirus, Haemophilus influenza type b, tuberculosis, measles, mumps, rubella, and herpes zoster. The types of industry conflict for the “industry-funded” SRs included study funding by a vaccine manufacturer (3/27, 11%), and in most cases direct employment of at least one author by a vaccine manufacturer (24/27, 89%). All but two of these SRs did not report a conflict of interest. Out of the non-industry funded SRs, 47% (14/30) reported no funding, while 43% (13/30) reported funding from non-profit organizations or governmental/national agencies. Further study characteristics are shown in Additional file 4.

### Methodological quality

**Fig. 2:**
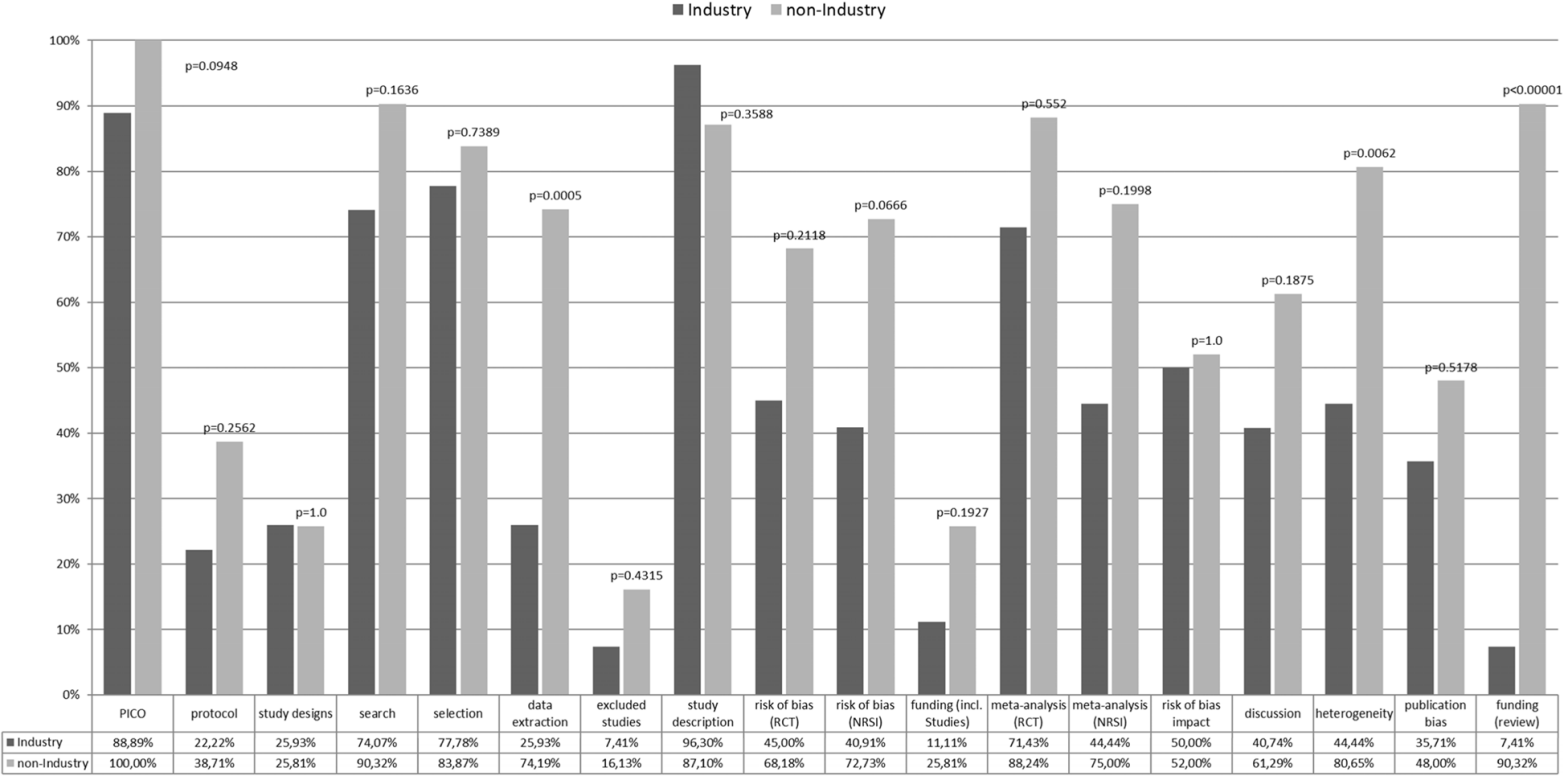
AMSTAR 2 ratings at item level for industry funded vs. non-industry funded SRs

The mean AMSTAR 2 summary score across all SRs was 0.49. Overall, the median AMSTAR 2 summary score was higher for the non-industry funded SRs than for the industry-funded SRs (0.62 vs. 0.36; *p* < .00001). Industry funded SRs were less likely to fulfill all but one AMSTAR 2 item compared to non-industry funded SRs (Fig. 2), though the difference was significant only for three specific items (item 6: data extraction, item 14: assessment of heterogeneity, and item 16: conflict of interest). All AMSTAR ratings can be found in Additional file 5.

## Discussion

This is the first study to focus on the influence of industry-funding on the methodological quality of vaccine SRs. We found that overall methodological quality of SRs of vaccines was suboptimal. Furthermore, industry-funded vaccine SRs over the last three years turned out to have a lower methodological quality than non-industry funded SRs.

The number of published SRs is rising steadily [9, 86]. In our sample, the number of published SRs was rather balanced across 2016 to 2018, while there was a notable increase in 2019. Of all SRs that met our eligibility criteria, 15% (27/185) were industry funded. This can be considered a high proportion of industry funded SRs. In two recent samples of SRs in dentistry the proportion of industry-funded SRs was 2% [87], while it was 3% in a general sample of SRs [88]. In a sample of SRs in vaccinology published between 1991 and 2007 the proportion of industry funded SRs was 7% (9/121) [89].

Overall, the methodological quality of the included SRs was low to moderate as indicated by a mean summary score of 0.49. We acknowledge that calculating a summary score is not the recommended approach to derive the methodological quality; nevertheless, others have already started using this intuitive approach [90]. Furthermore, for the purposes of statistical comparative research-on-research studies such as ours, this method allows for the comparison of two groups of SRs by ruling out potential floor effects that might arise with the original rating system [27]. Other studies that used a comparable approach to derive the methodological quality revealed even lower scores of 0.19 in rehabilitation [91], while it was only slightly higher with 0.55 in a sample of acupuncture SRs [92]. Interestingly, in contrast to our findings, an older study using AMSTAR (the original version preceding AMSTAR 2) found SRs on influenza vaccination to be of high methodological quality [93], while moderate methodological quality was recently found in a study dealing with SRs on interventions aimed at improving vaccination coverage [94].

To the best of our knowledge, the influence of industry funding in SRs on vaccines has only been investigated in two studies. Both studies were not specifically designed to analyze the impact of industry funding. In the first study, Remschmidt et al. focused on SRs on influenza vaccination only [93]. The median AMSTAR score was higher for non-industry funded SRs but the difference did not reach statistical significance. Our present sample of SRs was not restricted to any specific vaccine-preventable disease. One can speculate whether the difference in the samples between our broad analysis versus the targeted influenza analysis can explain the different findings. One potential explanation is that influenza vaccines require complex summative analyses across seasons, and the large impact of the seasonal influenza vaccine programs could drive a high quality effect. This would explain the high methodological quality of these SRs observed in the study by Remschmidt et al. [93]. In the second study, De Vito et al. analyzed characteristics and methodological quality of SRs in vaccinology. They found that financial support was associated with a higher methodological quality. However, they did not separate their analysis to investigate the influence of industry funding, although they reported that SRs obtaining for-profit funding had a slightly higher methodological quality than SRs with non-profit funding [89]. Overall, the comparability of this study might be hampered by the fact that they used the Overview Quality Assessment Questionnaire (OQAQ) to assess the methodological quality. The OQAQ was the only validated tool at the time of conducting the study [95], while it has been superseded by AMSTAR that draws on the OQAQ and lately AMSTAR 2 due to criticism and methodological improvements.

The other most commonly used critical appraisal tool for SRs is the Risk of Bias in Systematic Reviews (ROBIS) tool [96]. While ROBIS and AMSTAR have been found to be equally valid [22, 24, 25], it is notable that ROBIS does not ask for the source of funding at all. Our findings underscore the need to also consider source of funding.

Other studies we can compare our findings to do not focus on vaccines but on pharmaceuticals. Although there might be some differences between vaccines and pharmaceuticals given some challenges that are mostly unique to vaccine SRs, there is overlap in the manufacturers of vaccines and pharmaceuticals. Several studies found that industry-funded SRs had lower methodological quality and had more flaws in reporting [97–99], while a recently published small study did not [100]. One of these studies also highlighted that the quality of industry funded SRs improved over time [99].

At the item level, we found statistically significant differences for data extraction (item 6), assessment of heterogeneity (item 14), and conflict of interest (item 16). Item 6 and item 14 were not found to be a source of difference between industry and non-industry funded SRs in any of the other comparable studies. In particular, the difference in item 6 is somewhat surprising. However, not fulfilling item 6 does not necessarily mean that data extraction was not performed in duplicate but could also mean that this was not sufficiently reported. The developers of AMSTAR 2 have declared seven items to be critical [20]. However, none of the three items we found a difference for is critical according to the developers of AMSTAR 2. A recent survey involving a ranking exercise also found these three items not to be high-ranked when compared to the remaining AMSTAR 2 items [101]. We also experienced difficulties when assessing item 16 for industry-funded SRs. To fulfill item 16 on conflict of interest, SR authors either have to declare that there were no competing interests which is obviously not an option for industry funded SRs, or to describe their funding sources and how they managed potential conflicts of interest. However, we found no guidance on what constitutes an adequate way to manage conflict of interest in industry funded SRs. We only considered this to be fulfilled by two SRs what might have been very strict given that no guidance is available.

### Strength and limitations

Our study is able to provide an up-to-date picture of the number of methodological quality of SRs in the field of vaccination. However, we restricted our sample to SRs dealing with interventions only as AMSTAR 2 has been designed for this type of SRs only, while it has not been validated for other types of SRs (e.g., etiology/risk factors, prevalence or incidence). Thus, all but one SR (including a network meta-analysis) were evaluated with a measurement tool that has been validated for this purpose, what has been found a methodological flaw before [102]. We are not certain that our findings can be generalized and extrapolated to other types of SRs. It is also possible that we missed relevant SRs as some terms were only searched in the title. Furthermore, it is likely that AMSTAR 2 is not able to fully capture all methodological challenges inherent to SRs in this field. We generated an overall score for AMSTAR 2 and did not rely on the overall confidence. To ensure consistency, all steps of study selection, data extraction and critical appraisal involved a calibration exercise and the involvement of an experienced reviewer (DP). Thus, we are confident that the approach of combining the results of multiple reviewers did not hamper the quality of our data. We originally also intended to look at spin and association of industry funding with results but were unable due to feasibility issues. We admit that the protocol was not publicly available.

## Conclusion

The methodological quality of SRs in vaccination is comparable to SRs in other fields, while it is still suboptimal. Methodological quality of SRs needs to improve, which is a task for authors, reviewers, and editors. In particular, decision-makers relying on SRs to inform health policy should be aware of methodological limitations of SRs. We are not able to provide a satisfactory explanation why industry funded SRs had a lower methodological quality than non-industry funded SRs over recent years. This needs to be explored in future. Industry funding is an important indicator of methodological quality for vaccine SRs and should be carefully considered when appraising SR quality.

## Supplementary Information

**Additional file 1.** Study protocol.

**Additional file 2.** Search strategy.

**Additional file 3.** List of excluded studies.

**Additional file 4.** Characteristics of included SRs.

**Additional file 5.**

## Data Availability

All collected data can be found in the appendices.

## References

[1] Fernandes S, Jit M, Bozzani F, Griffiths UK, Scott JAG, Burchett HED (2018). A bibliometric analysis of systematic reviews on vaccines and immunisation. Vaccine..

[2] Farrington CP (1992). The measurement and interpretation of age-specific vaccine efficacy. Int J Epidemiol..

[3] Mori M, Oura A, Ohnishi H, Washio M (2008). Confounding in evaluating the effectiveness of influenza vaccine. Vaccine..

[4] Orenstein WA, Bernier RH, Dondero TJ, Hinman AR, Marks JS, Bart KJ (1985). Field evaluation of vaccine efficacy. Bull World Health Organ..

[5] Price D, Jefferson T, Demicheli V (2004). Methodological issues arising from systematic reviews of the evidence of safety of vaccines. Vaccine..

[6] Dimova RB, Egelebo CC, Izurieta HS (2020). Systematic review of published meta-analyses of vaccine safety. Stat Biopharmaceut Res.

[7] Adjagba A, MacDonald NE, Ortega-Pérez I, Duclos P (2017). Strengthening and sustainability of national immunization technical advisory groups (NITAGs) globally: Lessons and recommendations from the founding meeting of the global NITAG network. Vaccine..

[8] Duclos P (2010). National Immunization Technical Advisory Groups (NITAGs): guidance for their establishment and strengthening. Vaccine..

[9] Ioannidis JP (2016). The Mass production of redundant, misleading, and conflicted systematic reviews and meta-analyses. Milbank Q..

[10] Ge L, Tian JH, Li YN, Pan JX, Li G, Wei D (2018). Association between prospective registration and overall reporting and methodological quality of systematic reviews: a meta-epidemiological study. J Clin Epidemiol..

[11] Sun X, Zhou X, Zhang Y, Liu H (2019). Reporting and Methodological quality of systematic reviews and meta-analyses of nursing interventions in patients with Alzheimer’s disease: General Implications of the Findings. J Nurs Scholarsh..

[12] Sideri S, Papageorgiou SN, Eliades T (2018). Registration in the international prospective register of systematic reviews (PROSPERO) of systematic review protocols was associated with increased review quality. J Clin Epidemiol..

[13] Wasiak J, Tyack Z, Ware R, Goodwin N, Faggion CM (2017). Poor methodological quality and reporting standards of systematic reviews in burn care management. Int Wound J..

[14] Sharma S, Oremus M (2018). PRISMA and AMSTAR show systematic reviews on health literacy and cancer screening are of good quality. J Clin Epidemiol..

[15] Hansen C, Lundh A, Rasmussen K, Hróbjartsson A (2019). Financial conflicts of interest in systematic reviews: associations with results, conclusions, and methodological quality. Cochrane Database Syst Rev..

[16] Barnes DE, Bero LA (1998). Why review articles on the health effects of passive smoking reach different conclusions. Jama..

[17] Jadad AR, Moher M, Browman GP, Booker L, Sigouin C, Fuentes M (2000). Systematic reviews and meta-analyses on treatment of asthma: critical evaluation. BMJ..

[18] McGowan J, Sampson M, Salzwedel DM, Cogo E, Foerster V, Lefebvre C (2016). PRESS Peer Review of Electronic Search Strategies: 2015 Guideline Statement. J Clin Epidemiol..

[19] Drugs CAf, Health Ti. Strings attached: CADTH’s database search filters Ottawa, ON2016 [Available from: https://www.cadth.ca/resources/finding-evidence/strings-attached-cadths-database-search-filters.

[20] Shea BJ, Reeves BC, Wells G, Thuku M, Hamel C, Moran J (2017). AMSTAR 2: a critical appraisal tool for systematic reviews that include randomised or non-randomised studies of healthcare interventions, or both. Bmj..

[21] Shea BJ, Grimshaw JM, Wells GA, Boers M, Andersson N, Hamel C (2007). Development of AMSTAR: a measurement tool to assess the methodological quality of systematic reviews. BMC Med Res Methodol..

[22] Gates M, Gates A, Duarte G, Cary M, Becker M, Prediger B (2020). Quality and risk of bias appraisals of systematic reviews are inconsistent across reviewers and centers. J Clin Epidemiol..

[23] Leclercq V, Beaudart C, Tirelli E, Bruyère O (2020). Psychometric measurements of AMSTAR 2 in a sample of meta-analyses indexed in PsycINFO. J Clin Epidemiol..

[24] Lorenz RC, Matthias K, Pieper D, Wegewitz U, Morche J, Nocon M (2019). A psychometric study found AMSTAR 2 to be a valid and moderately reliable appraisal tool. J Clin Epidemiol..

[25] Pieper D, Puljak L, González-Lorenzo M, Minozzi S (2019). Minor differences were found between AMSTAR 2 and ROBIS in the assessment of systematic reviews including both randomized and nonrandomized studies. J Clin Epidemiol..

[26] Dettori JR, Skelly AC, Brodt ED (2020). Critically low confidence in the results produced by spine surgery systematic reviews: an AMSTAR-2 evaluation from 4 spine journals. Global Spine J..

[27] Lorenz RC, Matthias K, Pieper D, Wegewitz U, Morche J, Nocon M (2020). AMSTAR 2 overall confidence rating: lacking discriminating capacity or requirement of high methodological quality?. J Clin Epidemiol..

[28] Storman M, Storman D, Jasinska KW, Swierz MJ, Bala MM (2020). The quality of systematic reviews/meta-analyses published in the field of bariatrics: a cross-sectional systematic survey using AMSTAR 2 and ROBIS. Obes Rev..

[29] Bakker M, Bunge EM, Marano C, de Ridder M, De Moerlooze L (2016). Immunogenicity, effectiveness and safety of combined hepatitis A and B vaccine: a systematic literature review. Expert Rev Vaccines..

[30] Caspard H, Heikkinen T, Belshe RB, Ambrose CS (2016). A systematic review of the efficacy of live attenuated influenza vaccine upon revaccination of children. Hum Vaccin Immunother..

[31] Schiffner-Rohe J, Witt A, Hemmerling J, von Eiff C, Leverkus FW (2016). Efficacy of PPV23 in preventing pneumococcal pneumonia in adults at increased risk--a systematic review and meta-analysis. PLoS One..

[32] Bekkat-Berkani R, Wilkinson T, Buchy P, Dos Santos G, Stefanidis D, Devaster JM (2017). Seasonal influenza vaccination in patients with COPD: a systematic literature review. BMC Pulm Med..

[33] Cafiero-Fonseca ET, Stawasz A, Johnson ST, Sato R, Bloom DE (2017). The full benefits of adult pneumococcal vaccination: a systematic review. PLoS One..

[34] Caspard H, Mallory RM, Yu J, Ambrose CS (2017). Live-attenuated influenza vaccine effectiveness in children from 2009 to 2015-2016: a systematic review and meta-analysis. Open Forum. Infect Dis..

[35] Lansbury LE, Smith S, Beyer W, Karamehic E, Pasic-Juhas E, Sikira H (2017). Effectiveness of 2009 pandemic influenza A(H1N1) vaccines: a systematic review and meta-analysis. Vaccine..

[36] Sousa S, Duarte AC, Cordeiro I, Ferreira J, Goncalves MJ, Meirinhos T (2017). Efficacy and safety of vaccination in pediatric patients with systemic inflammatory rheumatic diseases: a systematic review of the literature. Acta Reumatol Port..

[37] Tin Tin Htar M, Stuurman AL, Ferreira G, Alicino C, Bollaerts K, Paganino C (2017). Effectiveness of pneumococcal vaccines in preventing pneumonia in adults, a systematic review and meta-analyses of observational studies. PLoS One..

[38] van den Ende C, Marano C, van Ahee A, Bunge EM, De Moerlooze L (2017). The immunogenicity of GSK's recombinant hepatitis B vaccine in children: a systematic review of 30 years of experience. Expert Rev Vaccines..

[39] Van Den Ende C, Marano C, Van Ahee A, Bunge EM, De Moerlooze L (2017). The immunogenicity and safety of GSK's recombinant hepatitis B vaccine in adults: a systematic review of 30 years of experience. Expert Rev Vaccines..

[40] Chit A, Zivaripiran H, Shin T, Lee JKH, Tomovici A, Macina D (2018). Acellular pertussis vaccines effectiveness over time: a systematic review, meta-analysis and modeling study. PLoS One..

[41] Dos Santos G, Tahrat H, Bekkat-Berkani R (2018). Immunogenicity, safety, and effectiveness of seasonal influenza vaccination in patients with diabetes mellitus: a systematic review. Hum Vaccin Immunother..

[42] Lee JKH, Lam GKL, Shin T, Kim J, Krishnan A, Greenberg DP (2018). Efficacy and effectiveness of high-dose versus standard-dose influenza vaccination for older adults: a systematic review and meta-analysis. Expert Rev Vaccines..

[43] Patterson J, Kagina BM, Gold M, Hussey GD, Muloiwa R (2018). Comparison of adverse events following immunisation with acellular and whole-cell pertussis vaccines: A systematic review. Vaccine..

[44] Steben M, Tan Thompson M, Rodier C, Mallette N, Racovitan V, DeAngelis F (2018). A review of the impact and effectiveness of the quadrivalent human papillomavirus vaccine: 10 years of clinical experience in Canada. J Obstet Gynaecol Can..

[45] Willame C, Vonk Noordegraaf-Schouten M, Gvozdenovic E, Kochems K, Oordt-Speets A, Praet N (2018). Effectiveness of the oral human attenuated rotavirus vaccine: a systematic review and meta-analysis-2006-2016. Open Forum. Infect Dis..

[46] Bravo C, Mege L, Vigne C, Thollot Y (2019). Clinical experience with the inactivated hepatitis A vaccine, Avaxim 80U Pediatric. Expert Rev Vaccines..

[47] D'Heilly C, Switzer C, Macina D (2019). Safety of maternal immunization against pertussis: a systematic review. Infect Dis Ther..

[48] Dolhain J, Janssens W, Sohn WY, Dindore V, Mukherjee P (2019). Integration of hexavalent diphtheria, tetanus, acellular pertussis, hepatitis B virus, inactivated poliomyelitis and Haemophilus influenzae type b conjugate vaccine within existing national recommendations following a birth dose of monovalent hepatitis B virus vaccine: results of a systematic review in the Asia Pacific region. Expert Rev Vaccines..

[49] McGirr A, Widenmaier R, Curran D, Espie E, Mrkvan T, Oostvogels L (2019). The comparative efficacy and safety of herpes zoster vaccines: a network meta-analysis. Vaccine..

[50] McLaughlin JM, Jiang Q, Gessner BD, Swerdlow DL, Sings HL, Isturiz RE (2019). Pneumococcal conjugate vaccine against serotype 3 pneumococcal pneumonia in adults: A systematic review and pooled analysis. Vaccine..

[51] Nieto Guevara J, Borys D, DeAntonio R, Guzman-Holst A, Hoet B. Interchangeability between pneumococcal conjugate vaccines for pediatric use: a systematic literature review. Expert Rev Vaccines. 2020;19(11):1011–22. 10.1080/14760584.2019.1688148. Epub 2019 Nov 21. 10.1080/14760584.2019.1688148 31751159

[52] Samson SI, Leventhal PS, Salamand C, Meng Y, Seet BT, Landolfi V (2019). Immunogenicity of high-dose trivalent inactivated influenza vaccine: a systematic review and meta-analysis. Expert Rev Vaccines..

[53] Sings HL, De Wals P, Gessner BD, Isturiz R, Laferriere C, McLaughlin JM (2019). Effectiveness of 13-valent pneumococcal conjugate vaccine against invasive disease caused by serotype 3 in children: a systematic review and meta-analysis of observational studies. Clin Infect Dis..

[54] Switzer C, D'Heilly C, Macina D (2019). Immunological and clinical benefits of maternal immunization against pertussis: a systematic review. Infect Dis Ther..

[55] Tin Tin Htar M, Sings HL, Syrochkina M, Taysi B, Hilton B, Schmitt HJ (2019). The impact of pneumococcal conjugate vaccines on serotype 19A nasopharyngeal carriage. Expert Rev Vaccines..

[56] de Oliveira LH, Camacho LA, Coutinho ES, Martinez-Silveira MS, Carvalho AF, Ruiz-Matus C (2016). Impact and effectiveness of 10 and 13-valent pneumococcal conjugate vaccines on hospitalization and mortality in children aged less than 5 years in Latin American countries: a systematic review. PLoS One..

[57] Ewald H, Briel M, Vuichard D, Kreutle V, Zhydkov A, Gloy V (2016). The clinical effectiveness of pneumococcal conjugate vaccines: a systematic review and meta-analysis of randomized controlled trials. Dtsch Arztebl Int..

[58] Liao Z, Tang H, Xu X, Liang Y, Xiong Y, Ni J (2016). Immunogenicity and safety of influenza vaccination in systemic lupus erythematosus patients compared with healthy controls: a meta-analysis. PLoS One..

[59] Nunes MC, Aqil AR, Omer SB, Madhi SA (2016). The effects of influenza vaccination during pregnancy on birth outcomes: a systematic review and meta-analysis. Am J Perinatol..

[60] Remschmidt C, Harder T, Wichmann O, Bogdan C, Falkenhorst G (2016). Effectiveness, immunogenicity and safety of 23-valent pneumococcal polysaccharide vaccine revaccinations in the elderly: a systematic review. BMC Infect Dis..

[61] Santos VS, Marques DP, Martins-Filho PR, Cuevas LE, Gurgel RQ (2016). Effectiveness of rotavirus vaccines against rotavirus infection and hospitalization in Latin America: systematic review and meta-analysis. Infect Dis Poverty..

[62] Azami M, Hafezi Ahmadi MR, Sayehmiri K (2017). Hepatitis B Vaccination efficacy in Iranian healthcare workers: a meta-analysis study. Hepat Mon..

[63] D'Addario M, Redmond S, Scott P, Egli-Gany D, Riveros-Balta AX, Henao Restrepo AM (2017). Two-dose schedules for human papillomavirus vaccine: systematic review and meta-analysis. Vaccine..

[64] Falkenhorst G, Remschmidt C, Harder T, Hummers-Pradier E, Wichmann O, Bogdan C (2017). Effectiveness of the 23-valent pneumococcal polysaccharide vaccine (PPV23) against pneumococcal disease in the elderly: systematic review and meta-analysis. PLoS One..

[65] Godoi IP, Lemos LL, de Araujo VE, Bonoto BC, Godman B, Guerra Junior AA (2017). CYD-TDV dengue vaccine: systematic review and meta-analysis of efficacy, immunogenicity and safety. J Comp Eff Res..

[66] Kassim P, Eslick GD (2017). Risk of intussusception following rotavirus vaccination: an evidence based meta-analysis of cohort and case-control studies. Vaccine..

[67] Lee KR, Bae JH, Hwang IC, Kim KK, Suh HS, Ko KD (2017). Effect of influenza vaccination on risk of stroke: a systematic review and meta-analysis. Neuroepidemiology..

[68] Ogawa Y, Takei H, Ogawa R, Mihara K (2017). Safety of human papillomavirus vaccines in healthy young women: a meta-analysis of 24 controlled studies. J Pharm Health Care Sci..

[69] Wilkinson K, Wei Y, Szwajcer A, Rabbani R, Zarychanski R, Abou-Setta AM (2017). Efficacy and safety of high-dose influenza vaccine in elderly adults: a systematic review and meta-analysis. Vaccine..

[70] Dzanibe S, Madhi SA (2018). Systematic review of the clinical development of group B streptococcus serotype-specific capsular polysaccharide-based vaccines. Expert Rev Vaccines..

[71] Genovese C, V LAF, Squeri A, Trimarchi G, Squeri R. (2018). HPV vaccine and autoimmune diseases: systematic review and meta-analysis of the literature. J Prev Med Hyg..

[72] Harder T, Wichmann O, Klug SJ, van der Sande MAB, Wiese-Posselt M (2018). Efficacy, effectiveness and safety of vaccination against human papillomavirus in males: a systematic review. BMC Med..

[73] Imai C, Toizumi M, Hall L, Lambert S, Halton K, Merollini K (2018). A systematic review and meta-analysis of the direct epidemiological and economic effects of seasonal influenza vaccination on healthcare workers. PLoS One..

[74] Mouchet J, Salvo F, Raschi E, Poluzzi E, Antonazzo IC, De Ponti F (2018). Human papillomavirus vaccine and demyelinating diseases-a systematic review and meta-analysis. Pharmacol Res..

[75] Xu X, Zhu H, Lv H (2018). Safety of Staphylococcus aureus four-antigen and three-antigen vaccines in healthy adults: a meta-analysis of randomized controlled trials. Hum Vaccin Immunother..

[76] Adetokunboh OO, Ndwandwe D, Awotiwon A, Uthman OA, Wiysonge CS (2019). Vaccination among HIV-infected, HIV-exposed uninfected and HIV-uninfected children: a systematic review and meta-analysis of evidence related to vaccine efficacy and effectiveness. Hum Vaccin Immunother..

[77] Caldeira D, Rodrigues B, David C, Costa J, Pinto FJ, Ferreira JJ (2019). The association of influenza infection and vaccine with myocardial infarction: systematic review and meta-analysis of self-controlled case series. Expert Rev Vaccines..

[78] García-Perdomo HA, Osorio JC, Fernandez A, Zapata-Copete JA, Castillo A (2019). The effectiveness of vaccination to prevent the papillomavirus infection: a systematic review and meta-analysis. Epidemiol Infect..

[79] Ji Z, Jian M, Chen T, Luo L, Li L, Dai X (2019). Immunogenicity and safety of the M72/AS01E candidate vaccine against tuberculosis: a meta-analysis. Front Immunol..

[80] Lindsey BB, Armitage EP, Kampmann B, de Silva TI (2019). The efficacy, effectiveness, and immunogenicity of influenza vaccines in Africa: a systematic review. Lancet Infect Dis..

[81] Mehtani NJ, Rosman L, Moss WJ (2019). immunogenicity and safety of the measles vaccine in HIV-infected children: an updated systematic review. Am J Epidemiol..

[82] Nic Lochlainn LM, de Gier B, van der Maas N, Strebel PM, Goodman T, van Binnendijk RS (2019). Immunogenicity, effectiveness, and safety of measles vaccination in infants younger than 9 months: a systematic review and meta-analysis. Lancet Infect Dis..

[83] Tan J, Xiong YQ, He Q, Liu YM, Wang W, Chen M (2019). Peri-conceptional or pregnancy exposure of HPV vaccination and the risk of spontaneous abortion: a systematic review and meta-analysis. BMC Pregnancy Childbirth..

[84] Wang A, Liu C, Wang Y, Yin A, Wu J, Zhang C (2019). Pregnancy outcomes after human papillomavirus vaccination in periconceptional period or during pregnancy: a systematic review and meta-analysis. Hum Vaccin Immunother..

[85] Sinzinger AX, Von Kries R, Siedler A, Wichmann O, Harder T (2020). Non-specific effects of MMR vaccines on infectious disease related hospitalizations during the second year of life in high-income countries: a systematic review and meta-analysis. Hum Vaccin Immunother..

[86] Fontelo P, Liu F (2018). A review of recent publication trends from top publishing countries. Syst Rev..

[87] Faggion CM, Atieh M, Zanicotti DG (2014). Reporting of sources of funding in systematic reviews in periodontology and implant dentistry. Br Dent J..

[88] Page MJ, Shamseer L, Altman DG, Tetzlaff J, Sampson M, Tricco AC (2016). Epidemiology and reporting characteristics of systematic reviews of biomedical research: a cross-sectional study. PLoS Med..

[89] De Vito C, Manzoli L, Marzuillo C, Anastasi D, Boccia A, Villari P (2007). A systematic review evaluating the potential for bias and the methodological quality of meta-analyses in vaccinology. Vaccine..

[90] Pieper D, Lorenz RC, Rombey T, Jacobs A, Rissling O, Freitag S (2021). Authors should clearly report how they derived the overall rating when applying AMSTAR 2-a cross-sectional study. J Clin Epidemiol..

[91] Dijkers MP, Akers KG, Dieffenbach S, Galen SS (2021). Systematic reviews of clinical benefits of exoskeleton use for gait and mobility in neurologic disorders: a tertiary study. Arch Phys Med Rehabil.

[92] Hung CY, Wu XY, Chung VC, Tang EC, Wu JC, Lau AY (2019). Overview of systematic reviews with meta-analyses on acupuncture in post-stroke cognitive impairment and depression management. Integr Med Res..

[93] Remschmidt C, Wichmann O, Harder T (2014). Methodological quality of systematic reviews on influenza vaccination. Vaccine..

[94] Jaca A, Ndze VN, Wiysonge CS (2019). Assessing the methodological quality of systematic reviews of interventions aimed at improving vaccination coverage using AMSTAR and ROBIS checklists. Hum Vaccin Immunother..

[95] Oxman AD, Guyatt GH (1991). Validation of an index of the quality of review articles. J Clin Epidemiol..

[96] Whiting P, Savović J, Higgins JP, Caldwell DM, Reeves BC, Shea B (2016). ROBIS: A new tool to assess risk of bias in systematic reviews was developed. J Clin Epidemiol..

[97] Jørgensen AW, Hilden J, Gøtzsche PC (2006). Cochrane reviews compared with industry supported meta-analyses and other meta-analyses of the same drugs: systematic review. Bmj..

[98] Jørgensen AW, Maric KL, Tendal B, Faurschou A, Gøtzsche PC (2008). Industry-supported meta-analyses compared with meta-analyses with non-profit or no support: differences in methodological quality and conclusions. BMC Med Res Methodol..

[99] Lane PW, Higgins JP, Anagnostelis B, Anzures-Cabrera J, Baker NF, Cappelleri JC (2013). Methodological quality of meta-analyses: matched-pairs comparison over time and between industry-sponsored and academic-sponsored reports. Res Synth Methods..

[100] Ferrell S, Demla S, Anderson JM, Weaver M, Torgerson T, Hartwell M (2022). Association between industry sponsorship and author conflicts of interest with outcomes of systematic reviews and meta-analyses of interventions for opioid use disorder. J Subst Abuse Treat..

[101] Leclercq V, Hiligsmann M, Parisi G, Beaudart C, Tirelli E, Bruyère O (2020). Best-worst scaling identified adequate statistical methods and literature search as the most important items of AMSTAR2 (A measurement tool to assess systematic reviews). J Clin Epidemiol.

[102] Pieper D, Koensgen N, Breuing J, Ge L, Wegewitz U (2018). How is AMSTAR applied by authors - a call for better reporting. BMC Med Res Methodol..

