# Supplementary material for: Impact of industry sponsorship on the quality of systematic reviews of vaccines: a cross-sectional analysis of studies published from 2016 to 2019
Source: Syst Rev. 2022 Aug 22;11:174. doi: 10.1186/s13643-022-02051-x (PMC9395849; doi:10.1186/s13643-022-02051-x)
Supplement: Supplementary file 1 — Additional file 1. Study protocol. [file 13643_2022_2051_MOESM1_ESM.docx]

Appendix 1: study protocol

**Protocol**

Impact of industry sponsorship on the quality of systematic reviews of vaccines: a cross-sectional analysis of studies published from 2016–2019

*First version finalized on 20/04/20*

**BACKGROUND**

Among decision makers, industry funding is often recognized as a potential source of bias for primary studies, where the design, conduct, analysis, and publication of the trial may be influenced to favour a product over others. This funding bias is also applicable to systematic reviews. As systematic reviews are increasingly leveraged to inform decision-making, the question of whether industry sponsorship impacts the risk of bias, results, and interpretation (“spin”) of systematic reviews remains and has not yet been empirically assessed in the vaccine field outside of influenza studies (Remschmidt et al, 2014).

**OBJECTIVE & RESEARCH QUESTION**

The objective of this study is to compare systematic reviews that were sponsored by industry, either through authorship, funding, or a combination thereof, and non-industry sources to evaluate whether industry sponsorship influences the risk of bias, results, interpretation, and publication status of systematic reviews of vaccines.

Therefore, the research question is as follows: Is industry sponsorship correlated with the risk of bias, results, and interpretation of systematic reviews of vaccines?

**INCLUSION CRITERIA**

**Types of studies**

- Systematic reviews of intervention studies (defined as having, at a minimum, performed a bibliographic database search and identified and selected relevant studies based on specified inclusion/exclusion criteria, with at least one included study)

**Types of participants**

- All populations
- All settings

**Types of interventions**

- One or more specific vaccine product (e.g., 13-valent pneumococcal conjugate vaccine) or a class of vaccine (e.g., pneumococcal vaccines)

**Types of outcome measures**

- Vaccine efficacy or effectiveness (defined as the reduction in the risk of an outcome occurring among individuals who received the vaccine of interest as compared with those given another vaccine or no vaccine)
- Immunogenicity (measured by vaccine-specific antibody response)
- Safety (defined as solicited/unsolicited local and/or systematic adverse events and serious adverse events)

**Others**

- The study is published between January 1, 2016 and December 31, 2019. The publication date restriction was chosen to facilitate the assessment of contemporary systematic reviews of vaccines in light of evolving systematic review methodology.

**EXCLUSION CRITERIA**

**Types of studies**

- Primary research
- Narrative reviews (including opinions and editorials), clinical practice guidelines, health technology assessment reports, and other types of reviews that do not meet the definition of systematic review provided in the inclusion criteria
- Systematic reviews of non-human vaccine studies, burden of vaccine-preventable disease, economic assessments of vaccination, risk assessments of vaccination, vaccine-related modelling, qualitative assessments of vaccination (e.g., opinion research), and vaccine program evaluation

**Interventions**

- Therapeutic vaccines

**Types of outcome measures**

- No relevant outcomes assessed

**Others**

- Duplicate
- Published before January 1, 2016 or after December 31, 2019
- The full text of the article is not available
- Funding information not available

**REVIEW METHODOLOGY**

**Search strategy**

- The search strategy will be developed in collaboration with a librarian from the Health Library of Health Canada and the Public Health Agency of Canada. The search strategy will be peer reviewed by another librarian from the Health Library.
- MEDLINE and EMBASE databases will be searched.
- The database searches will be restricted to identifying articles published between January 1, 2016 and December 31, 2019.
- No language restrictions will be imposed.

**Study selection**

- The search strategy will be recorded and detailed in a separate document.
- Articles retrieved from the database searches will be screened by title and abstract. The full text of articles deemed relevant based on inclusion and exclusion criteria, or that had insufficient information to exclude, will be retrieved and assessed for eligibility through full-text screening.
- Articles will be randomly ordered for full-text screening to facilitate the selection of a representative sample of eligible systematic reviews for cross-sectional analysis. As industry sponsored systematic reviews are likely to be underrepresented in the vaccine literature, industry sponsored systematic reviews will be over sampled. Therefore, articles meeting inclusion/exclusion criteria during full-text screening will be selected at a 1:1 ratio of industry sponsored and non-industry sponsored systematic reviews up to a total of 60 articles total per publication year for each year from 2016 to 2019.
- Study selection will be completed by a single reviewer after piloting of the study selection process for both title/abstract and full-text screening. A second reviewer will verify the screening decisions for excluded studies. Any disagreements in the screening decisions will be resolved by discussion and reaching a consensus. If no consensus can be reached, a third reviewer will be brought in to mediate.
- Reasons for exclusion will be documented by each reviewer.
- The review process will be performed using Microsoft Excel.

**Data extraction**

- Data from included articles will be extracted into Excel by each reviewer after piloting of data extraction fields and process. Afterwards, another reviewer will validate the accuracy of data extraction and conflicts will be resolved by discussion between the two reviewers. If no consensus can be reached, a third reviewer will be brought in to mediate.
- Data extracted will consist of variables under the following subheadings: study identifiers (i.e., author, country of the first/corresponding author, year of publication, journal, and Thomson Reuters impact factor of the journal), design (i.e., whether a database search was performed, the number of databases used, whether relevant studies were identified and selected based on specified inclusion/exclusion criteria, and whether methodological quality/risk of bias was appraised and with what quality appraisal tools), intervention (i.e., type of vaccine), outcome definition (i.e., efficacy, effectiveness, immunogenicity, and/or safety), types of studies included (i.e., clinical trials and/or observational studies), number of studies included, statistical analysis (i.e., whether meta-analysis was performed, whether heterogeneity was assessed, and whether publication bias was evaluated), industry sponsorship status (i.e., industry authorship and/or industry funding), policy partners (i.e., whether the systematic review was performed involving a policy partner), conflict of interest (i.e., declarations of industry-related conflict and how this conflict was managed), and key findings (i.e., narrative summary of the magnitude and statistical significance of review findings, methodological quality of included studies, and review conclusions).
- A data extraction form including all the proposed extraction variables will be created in Excel.
- Authors will be contacted in case of missing or unclear reporting of funding.

**Quality assessment**

- Following data extraction, a single reviewer will assess the methodological quality of the included systematic reviews using the AMSTAR 2 tool. Training and a calibration exercise for AMSTAR 2 will be carried out for the reviewers to maximize consistency in appraisal among the reviewers.
- A methodological quality appraisal form including all the AMSTAR 2 checklist items will be created in Excel.

**Data analysis plan**

- Chi-squared test will be used to compare single AMSTAR 2 domains between the industry sponsored and non-industry sponsored systematic reviews. Mann-Whitney U-test or Kruskal-Wallis test will be used to compare summary AMSTAR 2 scores. Summary AMSTAR 2 scores will be calculated by summing up the number of “yes” items divided by the number of all eligible all items. Items scored with “partial yes” will be scored with 0.5. Sub-analysis will be conducted to explore whether there are temporal trends in the correlations between industry sponsorship and the methodological quality of systematic reviews of vaccines.

**Protocol amendments**

- Therapeutic vaccines added as an exclusion criterion (25/04/2020)
- At least one included study in a SR (08/05/20)
