# Supplementary material for: Impact of industry sponsorship on the quality of systematic reviews of vaccines: a cross-sectional analysis of studies published from 2016 to 2019
Source: Syst Rev. 2022 Aug 22;11:174. doi: 10.1186/s13643-022-02051-x (PMC9395849; doi:10.1186/s13643-022-02051-x)
Supplement: Supplementary file 2 — Additional file 2. Search strategy. [file 13643_2022_2051_MOESM2_ESM.docx]

Appendix 2: search strategy

Database(s): **Embase**1974 to 2020 March 27**, Ovid MEDLINE(R) ALL**1946 to March 26, 2020
Search Strategy:

| **#** | **Searches** | **Results** |
| --- | --- | --- |
| 1 | (meta-analysis or systematic review).pt. or meta-analysis/ or systematic review/ or exp meta-analysis as topic/ or *"systematic reviews as topic"/ or exp technology assessment, biomedical/ | 594002 |
| 2 | ((systematic* adj3 (review* or overview*)) or (methodologic* adj3 (review* or overview*))).ti,ab,kf,kw. | 414077 |
| 3 | ((quantitative adj3 (review* or overview* or synthes*)) or (research adj3 (integrati* or overview*))).ti,ab,kf,kw. | 23332 |
| 4 | ((integrative adj3 (review* or overview*)) or (collaborative adj3 (review* or overview*)) or (pool* adj3 analy*)).ti,ab,kf,kw. | 63083 |
| 5 | (data synthes* or data extraction* or data abstraction*).ti,ab,kf,kw. | 58741 |
| 6 | (handsearch* or hand search*).ti,ab,kf,kw. | 20087 |
| 7 | (mantel haenszel or peto or der simonian or dersimonian or fixed effect* or latin square*).ti,ab,kf,kw. | 59553 |
| 8 | (met analy* or metanaly* or technology assessment* or HTA or HTAs or technology overview* or technology appraisal*).ti,ab,kf,kw. | 23995 |
| 9 | (meta regression* or metaregression*).ti,ab,kf,kw. | 19265 |
| 10 | (meta-analy* or metaanaly* or systematic review* or biomedical technology assessment* or bio-medical technology assessment*).mp,hw. | 772158 |
| 11 | (medline or cochrane or pubmed or medlars or embase or cinahl).ti,ab,hw. | 497019 |
| 12 | (cochrane or (health adj2 technology assessment) or evidence report).jw. | 45809 |
| 13 | (comparative adj3 (efficacy or effectiveness)).ti,ab,kf,kw. | 32489 |
| 14 | (outcomes research or relative effectiveness).ti,ab,kf,kw. | 22481 |
| 15 | ((indirect or indirect treatment or mixed-treatment) adj comparison*).ti,ab,kf,kw. | 6126 |
| 16 | or/1-15 [CADTH Systematic Reviews Hedge] | 1123546 |
| 17 | (vaccin* or immuniz* or immunis*).ti. | 395450 |
| 18 | exp *immunization/ or exp *vaccination/ or *Vaccination Refusal/ or *Anti-Vaccination Movement/ or *vaccines/ or *immunization programs/ | 230337 |
| 19 | or/17-18 [Vaccination] | 461349 |
| 20 | 16 and 19 | 7844 |
| 21 | limit 20 to yr="2016 - 2019" | 2889 |
| 22 | 21 use medall [Medline results] | 1159 |
| 23 | meta-analysis.pt. or exp meta analysis/ or "systematic review"/ or methodology/ or "meta analysis (topic)"/ or "systematic review (topic)"/ or biomedical technology assessment/ | 2195940 |
| 24 | ((systematic* adj3 (review* or overview*)) or (methodologic* adj3 (review* or overview*))).ti,ab,kw. | 412749 |
| 25 | ((quantitative adj3 (review* or overview* or synthes*)) or (research adj3 (integrati* or overview*))).ti,ab,kw. | 23274 |
| 26 | ((integrative adj3 (review* or overview*)) or (collaborative adj3 (review* or overview*)) or (pool* adj3 analy*)).ti,ab,kw. | 62999 |
| 27 | (data synthes* or data extraction* or data abstraction*).ti,ab,kw. | 58733 |
| 28 | (handsearch* or hand search*).ti,ab,kw. | 20087 |
| 29 | (mantel haenszel or peto or der simonian or dersimonian or fixed effect* or latin square*).ti,ab,kw. | 59525 |
| 30 | (met analy* or metanaly* or technology assessment* or HTA or HTAs or technology overview* or technology appraisal*).ti,ab,kw. | 23654 |
| 31 | (meta regression* or metaregression*).ti,ab,kw. | 19262 |
| 32 | (meta-analy* or metaanaly* or systematic review* or biomedical technology assessment* or bio-medical technology assessment*).mp,hw. | 772158 |
| 33 | (medline or cochrane or pubmed or medlars or embase or cinahl).ti,ab,hw. | 497019 |
| 34 | (cochrane or (health adj2 technology assessment) or evidence report).jw. | 45809 |
| 35 | (comparative adj3 (efficacy or effectiveness)).ti,ab,kw. | 31937 |
| 36 | (outcomes research or relative effectiveness).ti,ab,kw. | 21945 |
| 37 | ((indirect or indirect treatment or mixed-treatment) adj comparison*).ti,ab,kw. | 6049 |
| 38 | or/23-37 [CADTH Systematic Reviews Hedge] | 2713424 |
| 39 | (vaccin* or immuniz* or immunis*).ti. | 395450 |
| 40 | exp *vaccination/ or exp *immunization/ or exp *vaccine/ or *vaccine hesitancy/ or *vaccination refusal/ | 319291 |
| 41 | or/39-40 [Vaccination] | 488514 |
| 42 | 38 and 41 | 16511 |
| 43 | limit 42 to yr="2016 - 2019" | 2957 |
| 44 | 43 use oemezd [Embase results] | 1809 |
| 45 | or/22,44 | 2968 |
| 46 | remove duplicates from 45 | 1947 |
