# Supplementary material for: Impact of industry sponsorship on the quality of systematic reviews of vaccines: a cross-sectional analysis of studies published from 2016 to 2019
Source: Syst Rev. 2022 Aug 22;11:174. doi: 10.1186/s13643-022-02051-x (PMC9395849; doi:10.1186/s13643-022-02051-x)
Supplement: Supplementary file 3 — Additional file 3. List of excluded studies. [file 13643_2022_2051_MOESM3_ESM.docx]

Appendix 3: list of excluded studies

Reason for exclusion: focus

Bartoszko JJ, McNamara IF, Aras OAZ, Hylton DA, Zhang YB, Malhotra D, et al. Does consecutive influenza vaccination reduce protection against influenza: A systematic review and meta-analysis. Vaccine. 2018; 36: 3434-3444. http://dx.doi.org/10.1016/j.vaccine.2018.04.049.

Edouard S, Al-Tawfiq JA, Memish ZA, Yezli S, Gautret P. Impact of the Hajj on pneumococcal carriage and the effect of various pneumococcal vaccines. Vaccine. 2018; 36: 7415-7422. http://dx.doi.org/10.1016/j.vaccine.2018.09.017.

Rosettie KL, Vos T, Mokdad AH, Flaxman AD, Khalil I, Troeger C, et al. Indirect rotavirus vaccine effectiveness for the prevention of rotavirus hospitalization: A systematic review and meta-analysis. American Journal of Tropical Medicine and Hygiene. 2018; 98: 1197-1201. http://dx.doi.org/10.4269/ajtmh.17-0705.

Young B, Sadarangani S, Jiang L, Wilder-Smith A, Chen MIC. Duration of influenza vaccine effectiveness: A systematic review, meta-analysis, and meta-regression of test- negative design case-control studies. Journal of Infectious Diseases. 2018; 217: 731-741. http://dx.doi.org/10.1093/infdis/jix632.

Zhang W, Sun H, Atiquzzaman M, Sou J, Anis AH, Cooper C. Influenza vaccination for HIV-positive people: Systematic review and network meta-analysis. Vaccine. 2018; 36: 4077-4086. http://dx.doi.org/10.1016/j.vaccine.2018.05.077.

Reason for exclusion: language

Azami M, Nikpey S, Pakzad I, Sayehmiri K. Effects of immunization to hepatitis B vaccine in Iranian health staff: A systematic review and meta-analysis study. Koomesh. 2016; 17: 789-795.

Diez-Domingo J, Garces-Sanchez M, Gimenez-Sanchez F, Colomina-Rodriguez J, Martinon-Torres F. [What have we learnt about rotavirus in Spain in the last 10 years?]. Que hemos aprendido sobre rotavirus en Espana en los ultimos 10 anos? 2019; 91: 166-179. https://dx.doi.org/10.1016/j.anpedi.2019.01.024.

Ferahta N, Achek I, Dubourg J, Lang PO. Vaccines against Herpes zoster: Effectiveness, safety, and cost/benefit ratio. Presse Medicale. 2016; 45: 162-176. http://dx.doi.org/10.1016/j.lpm.2015.10.015.

Godoi IP, Nascimento RC, Lemos LL, Almeida AM, Acurcio FA, Guerra Jr AA. Efficacy, safety, and cost-effectiveness of 13-valent pneumococcal vaccine: Overview of the literature for incorporation purposes. Value in Health. 2016; 19: A212.

Guchev IA. Vaccinal Prevention of Lower Respiratory Tract Pneumococcal Disease in Adults Without Immunosuppression. Antibiotiki i khimioterapiia = Antibiotics and chemoterapy [sic]. 2016; 61: 48-57.

Meng Z, Zhang J, Zhang Z, Luo D, Yang X. Immunogenicity of inacitivated quadrivalent influenza vaccine in adults aged 18-64 years: A systematic review and Meta-analysis. Chinese Journal of Endemiology. 2018; 39: 1636-1641. http://dx.doi.org/10.3760/cma.j.issn.0254-6450.2018.12.019.

Mofrad MH, Jedi L, Ahmadi S. The role of human papilloma virus (HPV) vaccines in prevention of cervical cancer. Iranian Journal of Obstetrics, Gynecology and Infertility. 2016; 19: 22-29. http://dx.doi.org/10.22038/ijogi.2016.7667.

Tinoco M, Santos M, Reitor C, Rodrigues A, Monteiro J. Polysaccharide of escherichia coli in the prevention of recurrent urinary tract infection: An evidence-based review. Acta Medica Portuguesa. 2018; 31: 165-169. http://dx.doi.org/10.20344/amp.9367.

Reason for exclusion: publication year

Langedijk AC, De Pijper CA, Spijker R, Grobusch MP, Stijnis C. Rabies antibody response after booster immunization: A systematic review and meta-analysis. Tropical Medicine and International Health. 2017; 22: 357. http://dx.doi.org/10.1111/%28ISSN%291365-3156.

Mario D. Corrigendum to: Are the two human papillomavirus vaccines really similar? A systematic review of available evidence: Efficacy of the two vaccines against HPV (Journal of Immunology Research (2015) 2015 (435141) DOI: 10.1155/2015/435141). Journal of Immunology Research. 2017; 2017: 4583487. http://dx.doi.org/10.1155/2017/4583487.

Mousavi T, Moosazadeh M, Afshari M, Davoodi L, Haghshenas M. Efficacy of L1 protein vaccines against cervical and vaginal cancer: A systematic review and meta-analysis. Iranian Red Crescent Medical Journal. 2017; 19: e42732. http://dx.doi.org/10.5812/ircmj.42732.

Mulley WR, Le STT, Ives KE. Primary seroresponses to double-dose compared with standard-dose hepatitis B vaccination in patients with chronic kidney disease: A systematic review and meta-analysis. Nephrology Dialysis Transplantation. 2017; 32: 136-143. <http://dx.doi.org/10.1093/ndt/gfv443>.

Reason for exclusion: duplicate

Adawi M, Bragazzi NL, McGonagle D, Mahroum N, Damiani G, Bridgewood C, et al. The efficacy and safety of anti-pneumococcal vaccination in systemic lupus erythematosus patients: Systematic review and meta-analysis. Annals of the Rheumatic Diseases. 2019; 78: 1164. http://dx.doi.org/10.1136/annrheumdis-2019-eular.3488.

Akkoc G. Association between rotavirus vaccination and risk of intussusception among neonates and infants: A systematic review and meta-analysis. Cocuk Enfeksiyon Dergisi. 2019; 13: 217-225. http://dx.doi.org/10.5578/ced.201960.

Bryan S, Barbara C, Thomas J, Olaitan A. HPV vaccine in the treatment of usual type vulval and vaginal intraepithelial neoplasia: A systematic review. BJOG: An International Journal of Obstetrics and Gynaecology. 2019; 126: 239. http://dx.doi.org/10.1111/1471-0528.23-15703.

Bryan S, Barbara C, Thomas J, Olaitan A. HPV vaccine in the treatment of usual type vulval and vaginal intraepithelial neoplasia: A systematic review 11 Medical and Health Sciences 1107 Immunology 11 Medical and Health Sciences 1117 Public Health and Health Services. BMC Women's Health. 2019; 19: 3. http://dx.doi.org/10.1186/s12905-018-0707-9.

Izurieta HS, Chillarige Y, Kelman J, Wei Y, Lu Y, Xu W, et al. Relative effectiveness of cell-cultured and egg-based influenza vaccines among the U.S. elderly, 2018-19. Pharmacoepidemiology and Drug Safety. 2019; 28: 392. http://dx.doi.org/10.1002/pds.4864.

Lee JKH, Lam GKL, Shin T, Kim J, Krishnan A, Greenberg DP, et al. Efficacy and effectiveness of high-dose versus standard-dose influenza vaccination for older adults: a systematic review and meta-analysis. Expert Review of Vaccines. 2018; 17: 435-443. http://dx.doi.org/10.1080/14760584.2018.1471989.

Prattley S, New F, Geraghty R, Somani B. Role of vaccines for recurrent urinary tract infections: A systematic review. Journal of Urology. 2019; 201: e1053-e1054. http://dx.doi.org/10.1097/01.JU.0000557131.27790.26.

Sanz Fadrique R, Martin Arias L, Molina-Guarneros JA, Jimeno Bulnes N, Garcia Ortega P. Guillain-Barre syndrome and influenza vaccines: current evidence. Revista espanola de quimioterapia : publicacion oficial de la Sociedad Espanola de Quimioterapia. 2019; 32: 288-295.

Reason for exclusion: economical evaluation

Chongmelaxme B, Hammanee M, Phooaphirak W, Kotirum S, Hutubessy R, Chaiyakunapruk N. Economic evaluations of Haemophilus influenzae type b (Hib) vaccine: a systematic review. Journal of Medical Economics. 2017; 20: 1094-1106. http://dx.doi.org/10.1080/13696998.2017.1359181.

D'Angiolella LS, Lafranconi A, Cortesi PA, Rota S, Cesana G, Mantovani LG. Costs and effectiveness of influenza vaccination: a systematic review. Annali dell'Istituto superiore di sanita. 2018; 54: 49-57. http://dx.doi.org/10.4415/ANN_18_01_10.

de Boer PT, van Maanen BM, Damm O, Ultsch B, Dolk FCK, Crepey P, et al. A systematic review of the health economic consequences of quadrivalent influenza vaccination. Expert Review of Pharmacoeconomics and Outcomes Research. 2017; 17: 249-265. http://dx.doi.org/10.1080/14737167.2017.1343145.

Sartori AMC, Rozman LM, Decimoni TC, Leandro R, Novaes HMD, de Soarez PC. A systematic review of health economic evaluations of vaccines in Brazil. Human Vaccines and Immunotherapeutics. 2017; 13: 1454-1465. http://dx.doi.org/10.1080/21645515.2017.1282588.

Shields GE, Elvidge J, Davies LM. A systematic review of economic evaluations of seasonal influenza vaccination for the elderly population in the European Union. BMJ Open. 2017; 7: e014847. http://dx.doi.org/10.1136/bmjopen-2016-014847.

Suh J, Kim B, Yang Y, Suh DC, Kim E. Cost effectiveness of influenza vaccination in patients with acute coronary syndrome in Korea. Vaccine. 2017; 35: 2811-2817. http://dx.doi.org/10.1016/j.vaccine.2017.04.016.

Ting EEK, Sander B, Ungar WJ. Systematic review of the cost-effectiveness of influenza immunization programs. Vaccine. 2017; 35: 1828-1843. <http://dx.doi.org/10.1016/j.vaccine.2017.02.044>.

Reason for exclusion: congress abstracts, no full-text available

Azharuddin M, Adil M, Ghosh P, Kapur P, Sharma M. BCG vaccination against Mycobacterium tuberculosis infection in pediatrics: A systematic literature review and meta-analysis. Pharmacoepidemiology and Drug Safety. 2019; 28: 394. http://dx.doi.org/10.1002/pds.4864.

Aziminia N, Hadjipavlou M, Pandian S, Malde S, Hammadeh M. Vaccines for the prevention of recurrent urinary tract infections: A systematic review. European Urology, Supplements. 2018; 17: e327-e328.

Baay M, Bollaerts K, Verstraeten T. Safety of newly adjuvanted vaccines among older adults, a systematic literature review and meta-analysis. Pharmacoepidemiology and Drug Safety. 2018; 27: 11-12. http://dx.doi.org/10.1002/pds.4629.

Baker JM, Tate JE, Leon J, Haber MJ, Lopman BA. Assessing serum anti-rotavirus immunoglobulin a as a correlate of vaccine-induced protection against rotavirus gastroenteritis in high and low child mortality settings: Analysis of pooled in dividual-level data from nine clinical trials. American Journal of Tropical Medicine and Hygiene. 2019; 101: 70. http://dx.doi.org/10.4269/ajtmh.abstract2019.

Bloomer C, Sharif S, Khan R, Al Assaf N. Efficacy of intravenous immunoglobulins in the prophylaxis of infection in preterm and low birth weight neonates: A scientific literature review. Archives of Disease in Childhood. 2019; 104: A132-A133. http://dx.doi.org/10.1136/archdischild-2019-epa.306.

Chung J, Flannery B, Begue R, Caspard H, Demarcus L, Fowlkes A, et al. Individual patient-level data meta-analysis of live attenuated and inactivated influenza vaccine effectiveness among us children, 2013-2014 through 2015-2016. Open Forum Infectious Diseases. 2018; 5: S68. http://dx.doi.org/10.1093/ofid/ofy209.161.

Covington D, Kaydo S, Veley K. Hepatitis B virus (HBV) vaccine in pregnancy and impact on pregnancy outcome. Value in Health. 2018; 21: S151.

El-Matary W, Yap J, Gilmour S. Bacille Calmette-Guerin (BCG) vaccine for induction of remission in Crohn's disease. Cochrane Database of Systematic Reviews. 2019; 2019: CD008029. http://dx.doi.org/10.1002/14651858.CD008029.pub2.

Frederiksen JL. Vaccines and Optic Neuritis: A systematic review. European Journal of Neurology. 2018; 25: 256.

Fukuta H, Ohte N. The effect of influenza vaccination on mortality and hospitalization in patients with heart failure: A meta-analysis. Journal of the American College of Cardiology. 2018; 71http://dx.doi.org/10.1016/S0735-1097%2818%2931445-1.

Gasparini R, Tregnaghi M, Keshavan P, Ypma E, Han L, Smolenov I. Safety and Immunogenicity of a Quadrivalent Meningococcal Conjugate Vaccine and Commonly Administered Vaccines After Coadministration. The Pediatric infectious disease journal. 2016; 35: 81-93. https://dx.doi.org/10.1097/INF.0000000000000930.

Hamer MJ. Systematic review of marburg virus vaccine clinical trials. American Journal of Tropical Medicine and Hygiene. 2019; 101: 463. http://dx.doi.org/10.4269/ajtmh.abstract2019.

Hendrick L, Ward M. Pertussis vaccination: Should we be doing something different? Archives of Disease in Childhood. 2019; 104: A302-A303. http://dx.doi.org/10.1136/archdischild-2019-epa.711.

Kabanova A, Lilleri D. Analytic vaccinology: Antibody-driven design of a human cytomegalovirus subunit vaccine. Methods in Molecular Biology. 2016; 1403: 167-186. http://dx.doi.org/10.1007/978-1-4939-3387-7_8.

Kornetsky R, Greenberg D, Falup-Pecurariu O. Disparities in Effectiveness of Pneumococcal Vaccine in Industrialized and Developing Countries: Is Vaccination Closing the Gap? Antibiotiki i khimioterapiia = Antibiotics and chemoterapy [sic]. 2016; 61: 63-67.

Lee J, Lam G, Shin T, Kim J, Krishnan A, Seet B, et al. Efficacy and effectiveness of high-dose influenza vaccine for older adults: A systematic review and meta-analysis. Open Forum Infectious Diseases. 2017; 4: S456. http://dx.doi.org/10.1093/ofid/ofx163.1161.

Mangavelle J, Damin-Pernik M, Bellet F, Abadie D, Pageot C, Beyens MN. Acute posterior multifocal placoid pigment epitheliopathy after vaccination: Review of the literature and analysis of the French Pharmacovigilance database. Fundamental and Clinical Pharmacology. 2018; 32: 60. http://dx.doi.org/10.1111/fcp.12371.

McGirr A, Widenmaier R, Curran D, Espie E, Mrkvan T, Oostvogels L, et al. The comparative efficacy, safety, and reactogenicity, of herpes zoster vaccines: A network meta-analysis. Value in Health. 2018; 21: S149.

McNeil SA, Hatchette T, Andrew MK, Ambrose A, Boivin G, Diaz-Mitoma F, et al. Influenza vaccine effectiveness in the prevention of influenza-related hospitalization in Canadian adults over the 2011/12 through 2013/14 season: A pooled analysis from the serious outcomes surveillance (SOS) network of the Canadian influenza research network (CIRN). Open Forum Infectious Diseases. 2016; 3http://dx.doi.org/10.1093/ofid/ofw194.75.

Moore C, Gibbons F, Deiratany S, Okafor I, Cunney R, Drew R, et al. Potential impact of rotavirus vaccination on an Irish paediatric emergency department. European Journal of Pediatrics. 2016; 175: 1651. http://dx.doi.org/10.1007/s00431-016-2785-8.

Muusha P, Abdullahi L, Engel M. Human papillomavirus prevalence among women following HPV vaccine introduction: A systematic review. Sexually Transmitted Diseases. 2018; 45: S55.

Nowak O, Boronea B, John T, Shang J, Parihar H. Effectiveness of influenza vaccination in patients with diabetes: A systematic review. Journal of Managed Care and Specialty Pharmacy. 2018; 24: S39.

Okoli GN, Racovitan F, Righolt CH, Mahmud SM. Influence of comorbidity status on seasonal influenza vaccine effectiveness in Canada: A systematic review and metaanalysis of test-negative design studies. Pharmacoepidemiology and Drug Safety. 2019; 28: 387-388. http://dx.doi.org/10.1002/pds.4864.

Pool V, Mege L, Abou-Ali A. The risk of Arthus reaction following Tdap vaccination: Vaccine adverse event reporting system and a review of literature. Pharmacoepidemiology and Drug Safety. 2019; 28: 449-450. http://dx.doi.org/10.1002/pds.4864.

Prattley S, Geraghty R, Moore M, Somani BK. Role of Vaccines for Recurrent Urinary Tract Infections: A Systematic Review. European Urology Focus. 2019http://dx.doi.org/10.1016/j.euf.2019.11.002.

Saddier P, Marks MA, Calhoun S, Johnson K, Moride Y. Real-world effectiveness of the live zoster vaccine in preventing herpes zoster: A systematic review. Open Forum Infectious Diseases. 2018; 5: S743. http://dx.doi.org/10.1093/ofid/ofy210.2133.

Santos M, Tura BR, Silveira LT. Dengue vaccine efficacy: A systematic review. Value in Health. 2018; 21: S148-S149.

Sherif NA, Qureshi ZA, Alkomos MF, Alhusseiny AM, Elhusseiny KM, Mohyeldin IA, et al. Safety, efficacy and immunogenicity of dengue vaccines: Systematic review and network meta analysis of randomized controlled trials. American Journal of Tropical Medicine and Hygiene. 2018; 99: 508.

Syed O, Malde S, Sahai A. The role of vaccines in the prophylaxis of recurrent uncomplicated urinary tract infections in adult patients: A systematic reviewl. Neurourology and Urodynamics. 2018; 37: S361-S362.

Vanood A, Wingerchuk D. Systematic review investigating relationship between neuromyelitis optica spectrum disorder (NMOSD) and vaccination. Neurology. 2019; 92

Willame C, Henry O, Lin L, Baril L, Vetter V, Praet N. Measles, Mumps, and Rubella (MMR) vaccines differ considerably with regards to immediate injection pain: A systematic literature review. Pharmacoepidemiology and Drug Safety. 2016; 25: 522-523. http://dx.doi.org/10.1002/pds.4070.

Wulffraat N. Infectious risk and management of vaccination. Annals of the Rheumatic Diseases. 2019; 78: 3. http://dx.doi.org/10.1136/annrheumdis-2019-eular.8622.

Xu AY, Pang H. Meta-analysis of documents on vaccine effectiveness of live attenuated varicella vaccine in pupils and preschoolers. Chinese Journal of Biologicals. 2019; 32: 557-564.

Young B, Zhao X, Cook AR, Parry C, Wilder-Smith A, Chen M. Does the influenza vaccine provide year-round protection in the elderly? A systematic review and meta-analysis. Annals of the Academy of Medicine Singapore. 2016; 45: S344.

Reason for exclusion: no funding information

Baay M, Bollaerts K, Verstraeten T. A systematic review and meta-analysis on the safety of newly adjuvanted vaccines among older adults. Vaccine. 2018; 36: 4207-4214. http://dx.doi.org/10.1016/j.vaccine.2018.06.004.

Chong PP, Handler L, Weber DJ. A Systematic Review of Safety and Immunogenicity of Influenza Vaccination Strategies in Solid Organ Transplant Recipients. Clinical Infectious Diseases. 2018; 66: 1802-1811. http://dx.doi.org/10.1093/cid/cix1081.

Domnich A, Arata L, Amicizia D, Puig-Barbera J, Gasparini R, Panatto D. Effectiveness of MF59-adjuvanted seasonal influenza vaccine in the elderly: A systematic review and meta-analysis. Vaccine. 2017; 35: 513-520. http://dx.doi.org/10.1016/j.vaccine.2016.12.011.

Gkentzi D, Katsakiori P, Marangos M, Hsia Y, Amirthalingam G, Heath PT, et al. Maternal vaccination against pertussis: A systematic review of the recent literature. Archives of Disease in Childhood: Fetal and Neonatal Edition. 2017; 102: F456-F463. http://dx.doi.org/10.1136/archdischild-2016-312341.

Harder T, Koch J, Wichmann O, Hellenbrand W. Predicted vs observed effectiveness of outer membrane vesicle (OMV) vaccines against meningococcal serogroup B disease: Systematic review. Journal of Infection. 2017; 75: 81-94. http://dx.doi.org/10.1016/j.jinf.2017.05.001.

Helena De Oliveira L, Jauregui B, Carvalho AF, Giglio N. Impact and effectiveness of meningococcal vaccines: a review. Revista panamericana de salud publica = Pan American journal of public health. 2017; 41: e158. https://dx.doi.org/10.26633/RPSP.2017.158.

Jonesteller CL, Burnett E, Yen C, Tate JE, Parashar UD. Effectiveness of rotavirus vaccination: A systematic review of the first decade of global postlicensure data, 2006-2016. Clinical Infectious Diseases. 2017; 65: 840-850. http://dx.doi.org/10.1093/cid/cix369.

Katoh S, Suzuki M, Ariyoshi K, Morimoto K. Serotype replacement in adult pneumococcal pneumonia after the introduction of seven-valent pneumococcal conjugate vaccines for children in Japan: A systematic literature review and pooled data analysis. Japanese Journal of Infectious Diseases. 2017; 70: 495-501. http://dx.doi.org/10.7883/yoken.JJID.2016.311.

Koch J, Harder T, Von Kries R, Wichmann O. The risk of intussusception after rotavirus vaccination - A systematic literature review and meta-analysis. Deutsches Arzteblatt International. 2017; 114: 255-262. http://dx.doi.org/10.3238/arztebl.2017.0255.

Markowitz LE, Drolet M, Perez N, Jit M, Brisson M. Human papillomavirus vaccine effectiveness by number of doses: Systematic review of data from national immunization programs. Vaccine. 2018; 36: 4806-4815. http://dx.doi.org/10.1016/j.vaccine.2018.01.057.

Najafi F, Sayehmiri K, Najafi R. Efficacy of hepatitis B vaccination in under five-year-old children in Iran: A systematic review and meta-analysis study. Hepatitis Monthly. 2018; 18: e65385. http://dx.doi.org/10.5812/hepatmon.65385.

Sarkanen TO, Alakuijala APE, Dauvilliers YA, Partinen MM. Incidence of narcolepsy after H1N1 influenza and vaccinations: Systematic review and meta-analysis. Sleep Medicine Reviews. 2018; 38: 177-186. http://dx.doi.org/10.1016/j.smrv.2017.06.006.

Sestili C, Grazina I, La Torre G. HBV vaccine and risk of developing multiple sclerosis: a systematic review and meta-analysis. Human vaccines & immunotherapeutics. 2018https://dx.doi.org/10.1080/21645515.2018.1528835.

Signorelli C, Odone A, Ciorba V, Cella P, Audisio RA, Lombardi A, et al. Human papillomavirus 9-valent vaccine for cancer prevention: A systematic review of the available evidence. Epidemiology and Infection. 2017; 145: 1962-1982. http://dx.doi.org/10.1017/S0950268817000747.

Teo E, Lockhart K, Purchuri SN, Pushparajah J, Cripps AW, van Driel ML. Haemophilus influenzae oral vaccination for preventing acute exacerbations of chronic bronchitis and chronic obstructive pulmonary disease. Cochrane Database of Systematic Reviews. 2017; 2017: CD010010. http://dx.doi.org/10.1002/14651858.CD010010.pub3.

Zhang YY, Tang XF, Du CH, Wang BB, Bi ZW, Dong BR. Comparison of dual influenza and pneumococcal polysaccharide vaccination with influenza vaccination alone for preventing pneumonia and reducing mortality among the elderly: A meta-analysis. Human Vaccines and Immunotherapeutics. 2016; 12: 3056-3064. <http://dx.doi.org/10.1080/21645515.2016.1221552>.

Reason for exclusion: outcome

Alderfer J, Srivastava A, Isturiz R, Burman C, Absalon J, Beeslaar J, et al. Concomitant administration of meningococcal vaccines with other vaccines in adolescents and adults: a review of available evidence. Human Vaccines and Immunotherapeutics. 2019; 15: 2205-2216. http://dx.doi.org/10.1080/21645515.2019.1581542.

Allali S, Chalumeau M, Launay O, Ballas SK, de Montalembert M. Conjugate Haemophilus influenzae type b vaccines for sickle cell disease. Cochrane Database of Systematic Reviews. 2016; 2016: CD011199. http://dx.doi.org/10.1002/14651858.CD011199.pub2.

Balsells E, Dagan R, Yildirim I, Gounder PP, Steens A, Munoz-Almagro C, et al. The relative invasive disease potential of Streptococcus pneumoniae among children after PCV introduction: A systematic review and meta-analysis. Journal of Infection. 2018; 77: 368-378. http://dx.doi.org/10.1016/j.jinf.2018.06.004.

Bonetto C, Trotta F, Felicetti P, Alarcon GS, Santuccio C, Bachtiar NS, et al. Vasculitis as an adverse event following immunization - Systematic literature review. Vaccine. 2016; 34: 6641-6651. http://dx.doi.org/10.1016/j.vaccine.2015.09.026.

Kasting ML, Shapiro GK, Rosberger Z, Kahn JA, Zimet GD. Tempest in a teapot: A systematic review of HPV vaccination and risk compensation research. Human Vaccines and Immunotherapeutics. 2016; 12: 1435-1450. http://dx.doi.org/10.1080/21645515.2016.1141158.

Langedijk AC, De Pijper CA, Spijker R, Holman R, Grobusch MP, Stijnis C. Rabies antibody response after booster immunization: A Systematic Review and Meta-analysis. Clinical Infectious Diseases. 2018; 67: 1932-1947. http://dx.doi.org/10.1093/cid/ciy420.

Loubet P, Loulergue P, Galtier F, Launay O. Seasonal influenza vaccination of high-risk adults. Expert Review of Vaccines. 2016; 15: 1507-1518. http://dx.doi.org/10.1080/14760584.2016.1188696.

Madaras-Kelly K, Remington R, Hruza H, Xu D. Comparative effectiveness of high-dose versus standard-dose influenza vaccines in preventing postinfluenza deaths. Journal of Infectious Diseases. 2018; 218: 336-337. http://dx.doi.org/10.1093/infdis/jix645.

Muturi-Kioi V, Lewis D, Launay O, Leroux-Roels G, Anemona A, Loulergue P, et al. Neutropenia as an adverse event following vaccination: Results from randomized clinical trials in healthy adults and systematic review. PLoS ONE. 2016; 11: e0157385. http://dx.doi.org/10.1371/journal.pone.0157385.

Overduin LA, van Dongen JJM, Visser LG. The cellular immune response to rabies vaccination: A systematic review. Vaccines. 2019; 7: 110. http://dx.doi.org/10.3390/vaccines7030110.

Paillot R, El Hage CM. The use of a recombinant canarypox-based equine influenza vaccine during the 2007 Australian outbreak: A systematic review and summary. Pathogens. 2016; 5: 42. http://dx.doi.org/10.3390/pathogens5020042.

Preiss S, Chanthavanich P, Chen LH, Marano C, Buchy P, van Hoorn R, et al. Post-exposure prophylaxis (PEP) for rabies with purified chick embryo cell vaccine: a systematic literature review and meta-analysis. Expert Review of Vaccines. 2018; 17: 525-545. http://dx.doi.org/10.1080/14760584.2018.1473765.

Sakala IG, Eichinger KM, Petrovsky N. Neonatal vaccine effectiveness and the role of adjuvants. Expert Review of Clinical Immunology. 2019; 15: 869-878. http://dx.doi.org/10.1080/1744666X.2019.1642748.

Udomkarnjananun S, Takkavatakarn K, Praditpornsilpa K, Nader C, Eiam-Ong S, Jaber BL, et al. Hepatitis B virus vaccine immune response and mortality in dialysis patients: a meta-analysis. Journal of Nephrology. 2019http://dx.doi.org/10.1007/s40620-019-00668-1.

Valentino K, Poronsky CB. Human Papillomavirus Infection and Vaccination. Journal of pediatric nursing. 2016; 31: e155-e166. http://dx.doi.org/10.1016/j.pedn.2015.10.005.

van den Berg SPH, Warmink K, Borghans JAM, Knol MJ, van Baarle D. Effect of latent cytomegalovirus infection on the antibody response to influenza vaccination: a systematic review and meta-analysis. Medical Microbiology and Immunology. 2019; 208: 305-321. http://dx.doi.org/10.1007/s00430-019-00602-z.

Ventimiglia E, Horenblas S, Muneer A, Salonia A. Human Papillomavirus Infection and Vaccination in Males. European Urology Focus. 2016; 2: 355-362. <http://dx.doi.org/10.1016/j.euf.2016.08.012>.

Reason for exclusion: empty review

Odey F, Okomo U, Oyo-Ita A. Vaccines for preventing invasive salmonella infections in people with sickle cell disease. The Cochrane database of systematic reviews. 2018; 12: CD006975. http://dx.doi.org/10.1002/14651858.CD006975.pub4.

Poorolajal J, Hooshmand E. Booster dose vaccination for preventing hepatitis B. The Cochrane database of systematic reviews. 2016: CD008256. <http://dx.doi.org/10.1002/14651858.CD008256.pub3>.

Reason for exclusion: not a systematic review

Adetokunboh OO, Uthman OA, Wiysonge CS. Morbidity benefit conferred by childhood immunisation in relation to maternal HIV status: a meta-analysis of demographic and health surveys. Human Vaccines and Immunotherapeutics. 2018; 14: 2414-2426. http://dx.doi.org/10.1080/21645515.2018.1515453.

Beyer WEP, Palache AM, Boulfich M, Osterhaus ADME. Rationale for two influenza B lineages in seasonal vaccines: A meta-regression study on immunogenicity and controlled field trials. Vaccine. 2017; 35: 4167-4176. http://dx.doi.org/10.1016/j.vaccine.2017.06.038.

Butler AM, Layton JB, Dharnidharka VR, Sahrmann JM, Weber DJ, McGrath LJ. Comparative effectiveness of high dose versus standard-dose influenza vaccine among patients on chronic hemodialysis. Pharmacoepidemiology and Drug Safety. 2019; 28: 239. http://dx.doi.org/10.1002/pds.4864.

Cabrera A, Lepage JE, Sullivan KM, Seed SM. Vaxchora: A Single-Dose Oral Cholera Vaccine. Annals of Pharmacotherapy. 2017; 51: 584-589. http://dx.doi.org/10.1177/1060028017698162.

Chung JR, Flannery B, Ambrose CS, Begue RE, Caspard H, DeMarcus L, et al. Live attenuated and inactivated influenza vaccine effectiveness. Pediatrics. 2019; 143: e20182094. http://dx.doi.org/10.1542/peds.2018-2094.

Clark A, van Zandvoort K, Flasche S, Sanderson C, Bines J, Tate J, et al. Efficacy of live oral rotavirus vaccines by duration of follow-up: a meta-regression of randomised controlled trials. The Lancet. Infectious diseases. 2019http://dx.doi.org/10.1016/S1473-3099%2819%2930126-4.

Cohen PR. Injection Site Lichenoid Dermatitis Following Pneumococcal Vaccination: Report and Review of Cutaneous Conditions Occurring at Vaccination Sites. Dermatology and Therapy. 2016; 6: 287-298. http://dx.doi.org/10.1007/s13555-016-0105-x.

Costa APF, Cobucci RNO, Da Silva JM, Da Costa Lima PH, Giraldo PC, Goncalves AK. Safety of human papillomavirus 9-valent vaccine: A meta-analysis of randomized trials. Journal of Immunology Research. 2017; 2017: 3736201. http://dx.doi.org/10.1155/2017/3736201.

Curran D, Oostvogels L, Heineman T, Matthews S, McElhaney J, McNeil S, et al. Quality of Life Impact of an Adjuvanted Recombinant Zoster Vaccine in Adults Aged 50 Years and Older. The journals of gerontology. Series A, Biological sciences and medical sciences. 2019; 74: 1231-1238. http://dx.doi.org/10.1093/gerona/gly150.

de Gijsel D, von Reyn CF. A Breath of Fresh Air: BCG Prevents Adult Pulmonary Tuberculosis. International Journal of Infectious Diseases. 2019; 80: S6-S8. http://dx.doi.org/10.1016/j.ijid.2019.02.036.

Dermont MA, Elmer T. Influenza syndromic surveillance and vaccine efficacy in the UK Armed Forces, 2017-2018. Journal of the Royal Army Medical Corps. 2019; 165: 395-399. http://dx.doi.org/10.1136/jramc-2018-001067.

Fiorito TM, Baird GL, Alexander-Scott N, Bornschein S, Kelleher C, Du N, et al. Adverse Events Following Vaccination with Bivalent rLP2086 (Trumenba): An Observational, Longitudinal Study during a College Outbreak and a Systematic Review. Pediatric Infectious Disease Journal. 2018; 37: e13-e19. http://dx.doi.org/10.1097/INF.0000000000001742.

Garland SM, Kjaer SK, Munoz N, Block SL, Brown DR, Dinubile MJ, et al. Impact and effectiveness of the quadrivalent human papillomavirus vaccine: A systematic review of 10 years of real-world experience. Clinical Infectious Diseases. 2016; 63: 519-527. http://dx.doi.org/10.1093/cid/ciw354.

Hintze JM, O'Neill JP. Strengthening the case for gender-neutral and the nonavalent HPV vaccine. European Archives of Oto-Rhino-Laryngology. 2018; 275: 857-865. http://dx.doi.org/10.1007/s00405-018-4866-y.

Ishigami J, Sang Y, Grams ME, Coresh J, Chang A, Matsushita K. Effectiveness of Influenza Vaccination Among Older Adults Across Kidney Function: Pooled Analysis of 2005-2006 Through 2014-2015 Influenza Seasons. American Journal of Kidney Diseases. 2019http://dx.doi.org/10.1053/j.ajkd.2019.09.008.

Izurieta HS, Chillarige Y, Kelman J, Wei Y, Lu Y, Xu W, et al. Relative Effectiveness of Cell-Cultured and Egg-Based Influenza Vaccines among Elderly Persons in the United States, 2017-2018. Journal of Infectious Diseases. 2019; 220: 1255-1264. http://dx.doi.org/10.1093/infdis/jiy716.

James SF, Chahine EB, Sucher AJ, Hanna C. Shingrix: The New Adjuvanted Recombinant Herpes Zoster Vaccine. Annals of Pharmacotherapy. 2018; 52: 673-680. http://dx.doi.org/10.1177/1060028018758431.

Keitel WA, Voronca DC, Atmar RL, Paust S, Hill H, Wolff MC, et al. Effect of recent seasonal influenza vaccination on serum antibody responses to candidate pandemic influenza A/H5N1 vaccines: A meta-analysis. Vaccine. 2019; 37: 5535-5543. http://dx.doi.org/10.1016/j.vaccine.2019.04.066.

Kliner M, Keenan A, Sinclair D, Ghebrehewet S, Garner P. Influenza vaccination for healthcare workers in the UK: Appraisal of systematic reviews and policy options. BMJ Open. 2016; 6: e012149. http://dx.doi.org/10.1136/bmjopen-2016-012149.

Lamberti LM, Ashraf S, Walker CLF, Black RE. A systematic review of the effect of rotavirus vaccination on diarrhea outcomes among children younger than 5 years. Pediatric Infectious Disease Journal. 2016; 35: 992-998. http://dx.doi.org/10.1097/INF.0000000000001232.

Langedijk AC, van Aalst M, Meek B, van Leeuwen EMM, Zeerleder S, Meijer E, et al. Long-term pneumococcal vaccine immunogenicity following allogeneic hematopoietic stem cell transplantation. Vaccine. 2019; 37: 510-515. http://dx.doi.org/10.1016/j.vaccine.2018.11.053.

Levett-Jones T. Vaccines for preventing influenza in the elderly: A Cochrane review summary. International journal of nursing studies. 2019: 103372. https://dx.doi.org/10.1016/j.ijnurstu.2019.06.003.

Mbawuike IN, Atmar RL, Patel SM, Corry DB, Winokur PL, Brady RC, et al. Cell mediated immune responses following revaccination with an influenza A/H5N1 vaccine. Vaccine. 2016; 34: 547-554. http://dx.doi.org/10.1016/j.vaccine.2015.11.055.

McGirr A, Iqbal SM, Izurieta P, Talarico C, Luijken J, Redig J, et al. A systematic literature review and network meta-analysis feasibility study to assess the comparative efficacy and comparative effectiveness of pneumococcal conjugate vaccines. Human Vaccines and Immunotherapeutics. 2019; 15: 2713-2724. http://dx.doi.org/10.1080/21645515.2019.1612667.

Mensah VA, Gueye A, Ndiaye M, Edwards NJ, Wright D, Anagnostou NA, et al. Safety, immunogenicity and efficacy of prime-Boost vaccination with chad63 and mva encoding me-trap against plasmodium falciparum infection in adults in senegal. PLoS ONE. 2016; 11: e0167951. http://dx.doi.org/10.1371/journal.pone.0167951.

Moorlag SJCFM, Arts RJW, van Crevel R, Netea MG. Non-specific effects of BCG vaccine on viral infections. Clinical Microbiology and Infection. 2019; 25: 1473-1478. http://dx.doi.org/10.1016/j.cmi.2019.04.020.

Moreira ED, Jr., Block SL, Ferris D, Giuliano AR, Iversen O-E, Joura EA, et al. Safety Profile of the 9-Valent HPV Vaccine: A Combined Analysis of 7 Phase III Clinical Trials. Pediatrics. 2016; 138https://dx.doi.org/10.1542/peds.2015-4387.

Mori R. Human papillomavirus vaccines: Global versus Japanese situations. Acta Cytologica. 2016; 60: 37. http://dx.doi.org/10.1159/000446388.

Nelson KN, Wallace AS, Sodha SV, Daniels D, Dietz V. Assessing strategies for increasing urban routine immunization coverage of childhood vaccines in low and middle-income countries: A systematic review of peer-reviewed literature. Vaccine. 2016; 34: 5495-5503. http://dx.doi.org/10.1016/j.vaccine.2016.09.038.

Nicolay U, Heijnen E, Nacci P, Patriarca PA, Leav B. Immunogenicity of aIIV3, MF59-adjuvanted seasonal trivalent influenza vaccine, in older adults >=65 years of age: Meta-analysis of cumulative clinical experience. International Journal of Infectious Diseases. 2019; 85: S1-S9. http://dx.doi.org/10.1016/j.ijid.2019.03.026.

Oligbu G, Hsia Y, Folgori L, Collins S, Ladhani S. Pneumococcal conjugate vaccine failure in children: A systematic review of the literature. Vaccine. 2016; 34: 6126-6132. http://dx.doi.org/10.1016/j.vaccine.2016.10.050.

Porudominsky R, Gotuzzo EH. Yellow fever vaccine and risk of developing serious adverse events: a systematic review. Revista panamericana de salud publica = Pan American journal of public health. 2018; 42: e75. https://dx.doi.org/10.26633/RPSP.2018.75.

Razavi SM, Saeednejad M, Salamati P. Vaccination in Hajj: An Overview of the Recent Findings. International journal of preventive medicine. 2016; 7: 129. https://dx.doi.org/10.4103/2008-7802.195826.

Sacks HS. Review: Adjuvant recombinant subunit vaccine prevents herpes zoster more than live attenuated vaccine in adults >= 50 years. Annals of Internal Medicine. 2019; 170: JC14. http://dx.doi.org/10.7326/ACPJC-2019-170-4-014.

Schaumburg F, De Pijper CA, Grobusch MP. Intradermal travel vaccinations-when less means more. Travel Medicine and Infectious Disease. 2019; 28: 3-5. http://dx.doi.org/10.1016/j.tmaid.2019.03.007.

Stevens D, Foley S. The Urommune© sublingual vaccine for women with recurrent UTIs-initial experience. BJU International. 2016; 117: 13. http://dx.doi.org/10.1111/bju.13452.

Thomas RE. Yellow fever vaccine-associated viscerotropic disease: current perspectives. Drug design, development and therapy. 2016; 10: 3345-3353.

Vasileiou E, Sheikh A, Butler C, El Ferkh K, Simpson CR. Safety and effectiveness of influenza vaccines in people with asthma: A systematic review and meta-analysis. Thorax. 2016; 71: A155. http://dx.doi.org/10.1136/thoraxjnl-2016-209333.276.

Velazquez RF, Linhares AC, Munoz S, Seron P, Lorca P, DeAntonio R, et al. Efficacy, safety and effectiveness of licensed rotavirus vaccines: A systematic review and meta-analysis for Latin America and the Caribbean. BMC Pediatrics. 2017; 17: 14. http://dx.doi.org/10.1186/s12887-016-0771-y.

Young-Xu Y, Snider JT, van Aalst R, Mahmud SM, Thommes EW, Lee JKH, et al. Analysis of relative effectiveness of high-dose versus standard-dose influenza vaccines using an instrumental variable method. Vaccine. 2019; 37: 1484-1490. http://dx.doi.org/10.1016/j.vaccine.2019.01.063.

Zhu F, Deckx H, Roten R, Michiels B, Sarnecki M. Comparative Efficacy, Safety and Immunogenicity of Hepavax-Gene TF and Engerix-B Recombinant Hepatitis B Vaccines in Neonates in China. Pediatric Infectious Disease Journal. 2017; 36: 94-101. <http://dx.doi.org/10.1097/INF.0000000000001361>.

Reason for exclusion: reference to other publication

Arbyn M, Xu L. Efficacy and safety of prophylactic HPV vaccines. A Cochrane review of randomized trials. Expert Review of Vaccines. 2018; 17: 1085-1091. http://dx.doi.org/10.1080/14760584.2018.1548282.

Gemmill I, Young K. Summary of the NACI literature review on the comparative effectiveness of subunit and split virus inactivated influenza vaccines in older adults. Canada communicable disease report = Releve des maladies transmissibles au Canada. 2018; 44: 129-133.

Isenor JE, Bowles SK. Evidence for pharmacist vaccination. Canadian Pharmacists Journal. 2018; 151: 301-304. http://dx.doi.org/10.1177/1715163518783000.

Morrison J, Lasserson T. HPV vaccination: balancing facts. The Cochrane database of systematic reviews. 2018; 6: ED000126. <http://dx.doi.org/10.1002/14651858.ED000126>.

Reason for exclusion: study design

Luo H, Wang J, Yue F, Zhang C, Zhu J, Shao X. Effect of vaccination on heart failure patients: A systematic review and meta-analysis of observational studies. European Journal of Preventive Cardiology. 2018; 25: 1200-1201. http://dx.doi.org/10.1177/2047487318772931.

Alicino C, Paganino C, Orsi A, Astengo M, Trucchi C, Icardi G, et al. The impact of 10-valent and 13-valent pneumococcal conjugate vaccines on hospitalization for pneumonia in children: A systematic review and meta-analysis. Vaccine. 2017; 35: 5776-5785. http://dx.doi.org/10.1016/j.vaccine.2017.09.005.

Bissett SL, Godi A, Jit M, Beddows S. Seropositivity to non-vaccine incorporated genotypes induced by the bivalent and quadrivalent HPV vaccines: A systematic review and meta-analysis. Vaccine. 2017; 35: 3922-3929. http://dx.doi.org/10.1016/j.vaccine.2017.06.028.

Croce E, Hatz C, Jonker EF, Visser LG, Jaeger VK, Buhler S. Safety of live vaccinations on immunosuppressive therapy in patients with immune-mediated inflammatory diseases, solid organ transplantation or after bone-marrow transplantation - A systematic review of randomized trials, observational studies and case reports. Vaccine. 2017; 35: 1216-1226. http://dx.doi.org/10.1016/j.vaccine.2017.01.048.

Huang Y, Wang H, Tam WWS. Is rheumatoid arthritis associated with reduced immunogenicity of the influenza vaccination? A systematic review and meta-analysis. Current Medical Research and Opinion. 2017; 33: 1901-1908. http://dx.doi.org/10.1080/03007995.2017.1329140.

Loharikar A, Suragh TA, MacDonald NE, Balakrishnan MR, Benes O, Lamprianou S, et al. Anxiety-related adverse events following immunization (AEFI): A systematic review of published clusters of illness. Vaccine. 2018; 36: 299-305. http://dx.doi.org/10.1016/j.vaccine.2017.11.017.

Mahmood S, Shah KU, Khan TM. Immune Persistence After Infant Hepatitis-B Vaccination: A Systematic Review and Meta-Analysis. Scientific reports. 2018; 8: 12550. http://dx.doi.org/10.1038/s41598-018-30512-8.

Vargas KM, Koil A, Dehority W. Recurrent Sterile Abscesses After Immunization With Aluminum-Adjuvant Based Vaccines. Clinical Pediatrics. 2018; 57: 733-737. http://dx.doi.org/10.1177/0009922817728702.

Zhu S, Zeng F, Xia L, He H, Zhang J. Incidence rate of breakthrough varicella observed in healthy children after 1 or 2 doses of varicella vaccine: Results from a meta-analysis. American Journal of Infection Control. 2018; 46: e1-e7. <http://dx.doi.org/10.1016/j.ajic.2017.07.029>.

Reason for exclusion: intervention

Chiappini E, Petrolini C, Sandini E, Licari A, Pugni L, Mosca FA, et al. Update on vaccination of preterm infants: a systematic review about safety and efficacy/effectiveness. Proposal for a position statement by Italian Society of Pediatric Allergology and Immunology jointly with the Italian Society of Neonatology. Expert Review of Vaccines. 2019; 18: 523-545. http://dx.doi.org/10.1080/14760584.2019.1604230.

Church JA, Parker EP, Kirkpatrick BD, Grassly NC, Prendergast AJ. Interventions to improve oral vaccine performance: a systematic review and meta-analysis. The Lancet Infectious Diseases. 2019; 19: 203-214. http://dx.doi.org/10.1016/S1473-3099%2818%2930602-9.

Costa R, Zaman S, Sharpe S, Helenowski I, Shaw C, Han H, et al. A brief report of toxicity end points of HER2 vaccines for the treatment of patients with HER2+ breast cancer. Drug Design, Development and Therapy. 2019; 13: 309-316. http://dx.doi.org/10.2147/DDDT.S188925.

Dodangeh S, Daryani A, Sharif M, Aghayan SA, Pagheh AS, Sarvi S, et al. A systematic review on efficiency of microneme proteins to induce protective immunity against Toxoplasma gondii. European Journal of Clinical Microbiology and Infectious Diseases. 2019; 38: 617-629. http://dx.doi.org/10.1007/s10096-018-03442-6.

Faridnia R, Daryani A, Sarvi S, Sharif M, Kalani H. Vaccination against Toxoplasma gondii using rhoptry antigens: a systematic review. Comparative Immunology, Microbiology and Infectious Diseases. 2018; 59: 32-40. http://dx.doi.org/10.1016/j.cimid.2018.09.005.

Lim SG, Agcaoili J, De Souza NNA, Chan E. Therapeutic vaccination for chronic hepatitis B: A systematic review and meta-analysis. Journal of Viral Hepatitis. 2019; 26: 803-817. http://dx.doi.org/10.1111/jvh.13085.

Mukherjee N, Wheeler KM, Svatek RS. Bacillus Calmette-Guerin treatment of bladder cancer: A systematic review and commentary on recent publications. Current Opinion in Urology. 2019; 29: 181-188. http://dx.doi.org/10.1097/MOU.0000000000000595.

Oligbu G, Fallaha M, Pay L, Ladhani S. Risk of invasive pneumococcal disease in children with sickle cell disease in the era of conjugate vaccines: a systematic review of the literature. British Journal of Haematology. 2019; 185: 743-751. http://dx.doi.org/10.1111/bjh.15846.

Panozzo CA, Pourmalek F, Brauchli Pernus Y, Pileggi GS, Woerner A, Bonhoeffer J. Arthritis and arthralgia as an adverse event following immunization: A systematic literature review. Vaccine. 2019; 37: 372-383. http://dx.doi.org/10.1016/j.vaccine.2018.06.067.

Rondaan C, Furer V, Heijstek MW, Agmon-Levin N, Bijl M, Breedveld FC, et al. Efficacy, immunogenicity and safety of vaccination in adult patients with autoimmune inflammatory rheumatic diseases: A systematic literature review for the 2019 update of EULAR recommendations. RMD Open. 2019; 5: e001035. http://dx.doi.org/10.1136/rmdopen-2019-001035.

Rosenberg T, Philipsen BB, Mehlum CS, Dyrvig AK, Wehberg S, Chirila M, et al. Therapeutic use of the human papillomavirus vaccine on recurrent respiratory papillomatosis: A systematic review and meta-analysis. Journal of Infectious Diseases. 2019; 219: 1016-1425. http://dx.doi.org/10.1093/infdis/jiy616.

Silcock R, Crawford NW, Perrett KP. Subcutaneous nodules: an important adverse event following immunization. Expert Review of Vaccines. 2019; 18: 405-410. http://dx.doi.org/10.1080/14760584.2019.1586540.

Tin Tin Htar M, van Den Biggelaar AHJ, Sings H, Ferreira G, Moffatt M, Hall-Murray C, et al. The impact of routine childhood immunization with higher-valent pneumococcal conjugate vaccines on antimicrobial-resistant pneumococcal diseases and carriage: a systematic literature review. Expert Review of Vaccines. 2019; 18: 1069-1089. http://dx.doi.org/10.1080/14760584.2019.1676155.

Vadlamudi NK, Chen A, Marra F. Impact of the 13-valent pneumococcal conjugate vaccine among adults: A systematic review and meta-analysis. Clinical Infectious Diseases. 2019; 69: 34-49. http://dx.doi.org/10.1093/cid/ciy872.

Villanueva-Cabezas JP, Coppo MJC, Durr PA, McVernon J. Vaccine efficacy against Indonesian Highly Pathogenic Avian Influenza H5N1: systematic review and meta-analysis. Vaccine. 2017; 35: 4859-4869. http://dx.doi.org/10.1016/j.vaccine.2017.07.059.

Wu Z, Bao H, Yao J, Chen Y, Lu S, Li J, et al. Suitable hepatitis B vaccine for adult immunization in China: a systematic review and meta-analysis. Human Vaccines and Immunotherapeutics. 2019; 15: 220-227. <http://dx.doi.org/10.1080/21645515.2018.1509172>.

Reason for exclusion: randomly excluded (meeting eligibility criteria)

Aaby P, Ravn H, Fisker AB, Rodrigues A, Benn CS. Is diphtheria-tetanus-pertussis (DTP) associated with increased female mortality? A meta-analysis testing the hypotheses of sex-differential non-specific effects of DTP vaccine. Transactions of the Royal Society of Tropical Medicine and Hygiene. 2016; 110: 570-581. http://dx.doi.org/10.1093/trstmh/trw073.

Adawi M, Bragazzi NL, McGonagle D, Watad S, Mahroum N, Damiani G, et al. Immunogenicity, safety and tolerability of anti-pneumococcal vaccination in systemic lupus erythematosus patients: An evidence-informed and PRISMA compliant systematic review and meta-analysis. Autoimmunity Reviews. 2019; 18: 73-92. http://dx.doi.org/10.1016/j.autrev.2018.08.002.

Agarwal R, Wahid MH, Yausep OE, Angel SH, Lokeswara AW. The Immunogenicity and Safety of CYD-Tetravalent Dengue Vaccine (CYD-TDV) in Children and Adolescents: A Systematic Review. Acta medica Indonesiana. 2017; 49: 24-33.

Aggeletopoulou I, Davoulou P, Konstantakis C, Thomopoulos K, Triantos C. Response to hepatitis B vaccination in patients with liver cirrhosis. Reviews in Medical Virology. 2017; 27: e1942. http://dx.doi.org/10.1002/rmv.1942.

Almalki SSR. Circulating rotavirus g and p strains post rotavirus vaccination in eastern mediterranean region. Saudi Medical Journal. 2018; 39: 755-766. http://dx.doi.org/10.15537/smj.2018.6.21394.

Arbyn M, Xu L, Simoens C, Martin-Hirsch PPL. Prophylactic vaccination against human papillomaviruses to prevent cervical cancer and its precursors. Cochrane Database of Systematic Reviews. 2018; 2018: CD009069. http://dx.doi.org/10.1002/14651858.CD009069.pub3.

Ateudjieu J, Stoll B, Bisseck AC, Tembei AM, Genton B. Safety profile of the meningococcal conjugate vaccine (MenafrivacTM) in clinical trials and vaccination campaigns: a review of published studies. Human Vaccines and Immunotherapeutics. 2019http://dx.doi.org/10.1080/21645515.2019.1652041.

Aziminia N, Hadjipavlou M, Philippou Y, Pandian SS, Malde S, Hammadeh MY. Vaccines for the prevention of recurrent urinary tract infections: a systematic review. BJU International. 2019; 123: 753-768. http://dx.doi.org/10.1111/bju.14606.

Badawi A, Shering M, Rahman S, Lindsay LR. A systematic review and meta-analysis for the adverse effects, immunogenicity and efficacy of Lyme disease vaccines: Guiding novel vaccine development. Canadian journal of public health = Revue canadienne de sante publique. 2017; 108: e62-e70. http://dx.doi.org/10.17269/cjph.108.5728.

Badurdeen S, Marshall A, Daish H, Hatherill M, Berkley JA. Safety and Immunogenicity of Early Bacillus Calmette-Guerin Vaccination in Infants Who Are Preterm and/or Have Low Birth Weights: A Systematic Review and Meta-analysis. JAMA Pediatrics. 2019; 173: 75-85. http://dx.doi.org/10.1001/jamapediatrics.2018.4038.

Bauwens J, Saenz L-H, Reusser A, Kunzli N, Bonhoeffer J. Safety of Co-Administration Versus Separate Administration of the Same Vaccines in Children: A Systematic Literature Review. Vaccines. 2019; 8https://dx.doi.org/10.3390/vaccines8010012.

Belongia EA, Simpson MD, King JP, Sundaram ME, Kelley NS, Osterholm MT, et al. Variable influenza vaccine effectiveness by subtype: a systematic review and meta-analysis of test-negative design studies. The Lancet Infectious Diseases. 2016; 16: 942-951. http://dx.doi.org/10.1016/S1473-3099%2816%2900129-8.

Belongia EA, Skowronski DM, McLean HQ, Chambers C, Sundaram ME, De Serres G. Repeated annual influenza vaccination and vaccine effectiveness: review of evidence. Expert Review of Vaccines. 2017; 16: 723-736. http://dx.doi.org/10.1080/14760584.2017.1334554.

Bergman H, Buckley BS, Villanueva G, Petkovic J, Garritty C, Lutje V, et al. Comparison of different human papillomavirus (HPV) vaccine types and dose schedules for prevention of HPV-related disease in females and males. Cochrane Database of Systematic Reviews. 2019; 2019: CD013479. http://dx.doi.org/10.1002/14651858.CD013479.

Bi Q, Ferreras E, Pezzoli L, Legros D, Ivers LC, Digilio L, et al. Protection against cholera from killed whole-cell oral cholera vaccines: a systematic review and meta-analysis. The Lancet Infectious Diseases. 2017; 17: 1080-1088. http://dx.doi.org/10.1016/S1473-3099%2817%2930359-6.

Bigaeva E, Doorn E, Liu H, Hak E. Meta-Analysis on Randomized Controlled Trials of Vaccines with QS-21 or ISCOMATRIX Adjuvant: Safety and Tolerability. PloS one. 2016; 11: e0154757. http://dx.doi.org/10.1371/journal.pone.0154757.

Bitterman R, Eliakim-Raz N, Vinograd I, Zalmanovici Trestioreanu A, Leibovici L, Paul M. Influenza vaccines in immunosuppressed adults with cancer. Cochrane Database of Systematic Reviews. 2018; 2018: CD008983. http://dx.doi.org/10.1002/14651858.CD008983.pub3.

Bryan S, Barbara C, Thomas J, Olaitan A. HPV vaccine in the treatment of usual type vulval and vaginal intraepithelial neoplasia: a systematic review. BMC women's health. 2019; 19: 3. https://dx.doi.org/10.1186/s12905-018-0707-9.

Buckley BS, Henschke N, Bergman H, Skidmore B, Klemm EJ, Villanueva G, et al. Impact of vaccination on antibiotic usage: a systematic review and meta-analysis. Clinical Microbiology and Infection. 2019; 25: 1213-1225. http://dx.doi.org/10.1016/j.cmi.2019.06.030.

Burgess L, Southern KW. Pneumococcal vaccines for cystic fibrosis. Cochrane Database of Systematic Reviews. 2016; 2016: CD008865. http://dx.doi.org/10.1002/14651858.CD008865.pub4.

Campbell H, Gupta S, Dolan GP, Kapadia SJ, Singh AK, Andrews N, et al. Review of vaccination in pregnancy to prevent pertussis in early infancy. Journal of Medical Microbiology. 2018; 67: 1426-1456. http://dx.doi.org/10.1099/jmm.0.000829.

Chavers T, De Oliveira LH, Parashar UD, Tate JE. Post-licensure experience with rotavirus vaccination in Latin America and the Caribbean: a systematic review and meta-analysis. Expert Review of Vaccines. 2018; 17: 1037-1051. http://dx.doi.org/10.1080/14760584.2018.1541409.

Chen YC, Zhou JH, Tian JM, Li BH, Liu LH, Wei K. Adjuvanted-influenza vaccination in patients infected with HIV: a systematic review and meta-analysis of immunogenicity and safety. Human Vaccines and Immunotherapeutics. 2019http://dx.doi.org/10.1080/21645515.2019.1672492.

Chung JR, Flannery B, Thompson MG, Gaglani M, Jackson ML, Monto AS, et al. Seasonal effectiveness of live attenuated and inactivated influenza vaccine. Pediatrics. 2016; 137: e20153279. http://dx.doi.org/10.1542/peds.2015-3279.

Ciapponi A, Bardach A, Rey Ares L, Glujovsky D, Cafferata ML, Cesaroni S, et al. Sequential inactivated (IPV) and live oral (OPV) poliovirus vaccines for preventing poliomyelitis. Cochrane Database of Systematic Reviews. 2019; 2019: CD011260. http://dx.doi.org/10.1002/14651858.CD011260.pub2.

Ciapponi A, Lee A, Bardach A, Glujovsky D, Rey-Ares L, Luisa Cafferata M, et al. Interchangeability between Pneumococcal Conjugate Vaccines: A Systematic Review and Meta-Analysis. Value in Health Regional Issues. 2016; 11: 24-34. http://dx.doi.org/10.1016/j.vhri.2015.12.001.

Cristina G, vinCenza LF, Giuseppe T, Sabrina N, ra FS. HPV VACCINE AND NEUROLOGICAL DISEASES: SYSTEMATIC REVIEW AND META-ANALYSIS OF THE LITERATURE. Acta Medica Mediterranea. 2019; 35: 2203-2210. http://dx.doi.org/10.19193/0393-6384_2019_4_345.

Da Silveira LTC, Tura B, Santos M. Systematic review of dengue vaccine efficacy. BMC Infectious Diseases. 2019; 19: 750. http://dx.doi.org/10.1186/s12879-019-4369-5.

Dai X, Bai R, Jian M, Ji Z, Ding Z, Wang F, et al. Immunogenicity of different dosing schedules of the human live attenuate rotavirus vaccine (RV1) in infants and children: a meta-analysis. Human Vaccines and Immunotherapeutics. 2019; 15: 1228-1236. http://dx.doi.org/10.1080/21645515.2018.1537742.

Darvishian M, van den Heuvel ER, Bissielo A, Castilla J, Cohen C, Englund H, et al. Effectiveness of seasonal influenza vaccination in community-dwelling elderly people: an individual participant data meta-analysis of test-negative design case-control studies. The Lancet Respiratory Medicine. 2017; 5: 200-211. http://dx.doi.org/10.1016/S2213-2600%2817%2930043-7.

Demicheli V, Jefferson T, Ferroni E, Rivetti A, Di Pietrantonj C. Vaccines for preventing influenza in healthy adults. Cochrane Database of Systematic Reviews. 2018; 2018: CD001269. http://dx.doi.org/10.1002/14651858.CD001269.pub6.

Diao WQ, Shen N, Yu PX, Liu BB, He B. Efficacy of 23-valent pneumococcal polysaccharide vaccine in preventing community-acquired pneumonia among immunocompetent adults: A systematic review and meta-analysis of randomized trials. Vaccine. 2016; 34: 1496-1503. http://dx.doi.org/10.1016/j.vaccine.2016.02.023.

Dion GR, Teng S, Boyd LR, Northam A, Mason-Apps C, Vieira D, et al. Adjuvant human papillomavirus vaccination for secondary prevention: A systematic review. JAMA Otolaryngology - Head and Neck Surgery. 2017; 143: 614-622. http://dx.doi.org/10.1001/jamaoto.2016.4736.

Dong R, Yang YF, Chen G, Shen Z, Zheng S. Risk of intussusception after rotavirus vaccination: A meta-analysis. International Journal of Clinical and Experimental Medicine. 2016; 9: 1306-1313.

Dutoit V, Migliorini D, Dietrich PY. Current strategies for vaccination in glioblastoma. Current Opinion in Oncology. 2019; 31: 514-521. http://dx.doi.org/10.1097/CCO.0000000000000575.

Elwood JM, Ameratunga R. Autoimmune diseases after hepatitis B immunization in adults: Literature review and meta-analysis, with reference to 'autoimmune/autoinflammatory syndrome induced by adjuvants' (ASIA). Vaccine. 2018; 36: 5796-5802. http://dx.doi.org/10.1016/j.vaccine.2018.07.074.

Fabrizi F, Maria Donato F, Messa P. Efficacy and safety of reinforced versus standard vaccine schedule towards hepatitis B in chronic kidney disease: A systematic review and meta-analysis. Hepatitis Monthly. 2017; 17: e44179. http://dx.doi.org/10.5812/hepatmon.44179.

Fadrique RS, Arias LM, Molina-Guarneros JA, Bulnes NJ, Ortega PG. Guillain-barre syndrome and influenza vaccines: Current evidence. Revista Espanola de Quimioterapia. 2019; 32: 288-295.

Feng S, Cowling BJ, Sullivan SG. Influenza vaccine effectiveness by test-negative design - Comparison of inpatient and outpatient settings. Vaccine. 2016; 34: 1672-1679. http://dx.doi.org/10.1016/j.vaccine.2016.02.039.

Flacco ME, Manzoli L, Rosso A, Marzuillo C, Bergamini M, Stefanati A, et al. Immunogenicity and safety of the multicomponent meningococcal B vaccine (4CMenB) in children and adolescents: a systematic review and meta-analysis. The Lancet Infectious Diseases. 2018; 18: 461-472. http://dx.doi.org/10.1016/S1473-3099%2818%2930048-3.

Fortanier AC, Venekamp RP, Boonacker CWB, Hak E, Schilder AGM, Sanders EAM, et al. Pneumococcal conjugate vaccines for preventing acute otitis media in children. Cochrane Database of Systematic Reviews. 2019; 2019: CD001480. http://dx.doi.org/10.1002/14651858.

Fukuta H, Goto T, Wakami K, Kamiya T, Ohte N. The effect of influenza vaccination on mortality and hospitalization in patients with heart failure: a systematic review and meta-analysis. Heart Failure Reviews. 2019; 24: 109-114. http://dx.doi.org/10.1007/s10741-018-9736-6.

Fulton TR, Phadke VK, Orenstein WA, Hinman AR, Johnson WD, Omer SB. Protective Effect of Contemporary Pertussis Vaccines: A Systematic Review and Meta-analysis. Clinical Infectious Diseases. 2016; 62: 1100-1110. http://dx.doi.org/10.1093/cid/ciw051.

Furuta M, Sin J, Ng ESW, Wang K. Efficacy and safety of pertussis vaccination for pregnant women - a systematic review of randomised controlled trials and observational studies. BMC Pregnancy and Childbirth. 2017; 17: 390. http://dx.doi.org/10.1186/s12884-017-1559-2.

Gagliardi AM, Andriolo BN, Torloni MR, Soares BG. Vaccines for preventing herpes zoster in older adults. Cochrane Database of Systematic Reviews. 2016; 2016: CD008858. http://dx.doi.org/10.1002/14651858.CD008858.pub3.

Gallagher T, Lipsitch M. Post-Exposure Effects of Vaccines on Infectious Diseases. Epidemiologic reviews. 2019http://dx.doi.org/10.1093/epirev/mxz014.

Garcia Garrido HM, Veurink AM, Leeflang M, Spijker R, Goorhuis A, Grobusch MP. Hepatitis A vaccine immunogenicity in patients using immunosuppressive drugs: A systematic review and meta-analysis. Travel Medicine and Infectious Disease. 2019; 32: 101479. http://dx.doi.org/10.1016/j.tmaid.2019.101479.

Garmpi A, Damaskos C, Garmpis N, Patsouras A, Savvanis S, Gravvanis N, et al. Pneumococcal vaccination strategies among HIV-infected adult patients: A review of the literature. In Vivo. 2019; 33: 1425-1430. http://dx.doi.org/10.21873/invivo.11620.

Ghozy S, Nam NH, Radwan I, Karimzadeh S, Tieu TM, Hashan MR, et al. Therapeutic efficacy of hepatitis B virus vaccine in treatment of chronic HBV infections: A systematic review and meta-analysis. Reviews in Medical Virology. 2019: e2089. http://dx.doi.org/10.1002/rmv.2089.

Giles ML, Krishnaswamy S, Macartney K, Cheng A. The safety of inactivated influenza vaccines in pregnancy for birth outcomes: a systematic review. Human Vaccines and Immunotherapeutics. 2019; 15: 687-699. http://dx.doi.org/10.1080/21645515.2018.1540807.

Harmala S, Parisinos CA, Shallcross L, O'Brien A, Hayward A. Effectiveness of influenza vaccines in adults with chronic liver disease: A systematic review and meta-analysis. BMJ Open. 2019; 9: e031070. http://dx.doi.org/10.1136/bmjopen-2019-031070.

Hauser MI, Muscatello DJ, Soh ACY, Dwyer DE, Turner RM. An indirect comparison meta-analysis of AS03 and MF59 adjuvants in pandemic influenza A(H1N1)pdm09 vaccines. Vaccine. 2019; 37: 4246-4255. http://dx.doi.org/10.1016/j.vaccine.2019.06.039.

Henderson M, Bragg A, Fahim G, Shah M, Hermes-DeSantis ER. A review of the safety and efficacy of vaccines as prophylaxis for clostridium difficile infections. Vaccines. 2017; 5: 25. http://dx.doi.org/10.3390/vaccines5030025.

Higgins JPT, Soares-Weiser K, Lopez-Lopez JA, Kakourou A, Chaplin K, Christensen H, et al. Association of BCG, DTP, and measles containing vaccines with childhood mortality: Systematic review. BMJ (Online). 2016; 355: i5170. http://dx.doi.org/10.1136/bmj.i5170.

Hungerford D, Smith K, Tucker A, Iturriza-Gomara M, Vivancos R, McLeonard C, et al. Population effectiveness of the pentavalent and monovalent rotavirus vaccines: A systematic review and meta-analysis of observational studies. BMC Infectious Diseases. 2017; 17: 569. http://dx.doi.org/10.1186/s12879-017-2613-4.

Jaiswal N, Singh S, Agarwal A, Chauhan A, Thumburu KK, Kaur H, et al. Equivalent schedules of intradermal fractional dose versus intramuscular full dose of inactivated polio vaccine for prevention of poliomyelitis. Cochrane Database of Systematic Reviews. 2019; 2019: CD011780. http://dx.doi.org/10.1002/14651858.CD011780.pub2.

Jefferson T, Rivetti A, Di Pietrantonj C, Demicheli V. Vaccines for preventing influenza in healthy children. Cochrane Database of Systematic Reviews. 2018; 2018: CD004879. http://dx.doi.org/10.1002/14651858.CD004879.pub5.

Jeong S, Jang EJ, Jo J, Jang S. Effects of maternal influenza vaccination on adverse birth outcomes: A systematic review and Bayesian meta-analysis. PLoS ONE. 2019; 14: e0220910. http://dx.doi.org/10.1371/journal.pone.0220910.

Jiang HY, Shi YD, Zhang X, Pan LY, Xie YR, Jiang CM, et al. Human papillomavirus vaccination and the risk of autoimmune disorders: A systematic review and meta-analysis. Vaccine. 2019; 37: 3031-3039. http://dx.doi.org/10.1016/j.vaccine.2019.04.049.

Kandasamy R, Voysey M, McQuaid F, De Nie K, Ryan R, Orr O, et al. Non-specific immunological effects of selected routine childhood immunisations: Systematic review. BMJ (Online). 2016; 355: i5225. http://dx.doi.org/10.1136/bmj.i5225.

Kashangura R, Jullien S, Garner P, Johnson S. MVA85A vaccine to enhance BCG for preventing tuberculosis. The Cochrane database of systematic reviews. 2019; 4: CD012915. https://dx.doi.org/10.1002/14651858.CD012915.pub2.

Kessels J, Tarantola A, Salahuddin N, Blumberg L, Knopf L. Rabies post-exposure prophylaxis: A systematic review on abridged vaccination schedules and the effect of changing administration routes during a single course. Vaccine. 2019; 37: A107-A117. http://dx.doi.org/10.1016/j.vaccine.2019.01.041.

Kopsaftis Z, Wood-Baker R, Poole P. Influenza vaccine for chronic obstructive pulmonary disease (COPD). Cochrane Database of Systematic Reviews. 2018; 2018: CD002733. http://dx.doi.org/10.1002/14651858.CD002733.pub3.

Kraicer-Melamed H, O'Donnell S, Quach C. The effectiveness of pneumococcal polysaccharide vaccine 23 (PPV23) in the general population of 50 years of age and older: A systematic review and meta-analysis. Vaccine. 2016; 34: 1540-1550. http://dx.doi.org/10.1016/j.vaccine.2016.02.024.

La Torre G, Mannocci A, Colamesta V, D'Egidio V, Sestili C, Spadea A. Influenza and pneumococcal vaccination in hematological malignancies: A systematic review of efficacy, effectiveness, and safety. Mediterranean Journal of Hematology and Infectious Diseases. 2016; 8: e2016044. http://dx.doi.org/10.4084/MJHID.2016.044.

Lai JJ, Lin C, Ho CL, Chen PH, Lee CH. Alternative-dose versus standard-dose trivalent influenza vaccines for immunocompromised patients: A meta-analysis of randomised control trials. Journal of Clinical Medicine. 2019; 8: 590. http://dx.doi.org/10.3390/jcm8050590.

Lall D, Cason E, Pasquel FJ, Ali MK, Narayan KMV. Effectiveness of Influenza Vaccination for Individuals with Chronic Obstructive Pulmonary Disease (COPD) in Low-and Middle-Income Countries. COPD: Journal of Chronic Obstructive Pulmonary Disease. 2016; 13: 93-99. http://dx.doi.org/10.3109/15412555.2015.1043518.

Latifi-Navid H, Latifi-Navid S, Mostafaiy B, Jamalkandi SA, Ahmadi A. Pneumococcal Disease and the Effectiveness of the PPV23 Vaccine in Adults: A Two-Stage Bayesian Meta-Analysis of Observational and RCT Reports. Scientific reports. 2018; 8: 11051. http://dx.doi.org/10.1038/s41598-018-29280-2.

LeBras M, Barry A. Influenza vaccination for secondary cardiovascular prevention: A systematic review. Canadian Journal of Cardiology. 2016; 32: S112.

Leung VK, Cowling BJ, Feng S, Sullivan SG. Concordance of interim and final estimates of influenza vaccine effectiveness: A systematic review. Eurosurveillance. 2016; 21http://dx.doi.org/10.2807/1560-7917.ES.2016.21.16.30202.

Liao Z, Tang H, Xu X, Liang Y, Xiong Y, Ni J. Immunogenicity and Safety of Influenza Vaccination in Systemic Lupus Erythematosus Patients Compared with Healthy Controls: A Meta-Analysis. PLoS ONE. 2016; 11: e0147856. http://dx.doi.org/10.1371/journal.pone.0147856.

Loevinsohn G, Rosman L, Moss WJ. Measles Seroprevalence and Vaccine Responses in Human Immunodeficiency Virus-infected Adolescents and Adults: A Systematic Review. Clinical Infectious Diseases. 2019; 69: 836-844. http://dx.doi.org/10.1093/cid/ciy980.

Lopez AL, Deen J, Azman AS, Luquero FJ, Kanungo S, Dutta S, et al. Immunogenicity and Protection from a Single Dose of Internationally Available Killed Oral Cholera Vaccine: A Systematic Review and Metaanalysis. Clinical Infectious Diseases. 2018; 66: 1960-1971. http://dx.doi.org/10.1093/cid/cix1039.

Lu HL, Ding Y, Goyal H, Xu HG. Association between Rotavirus Vaccination and Risk of Intussusception among Neonates and Infants: A Systematic Review and Meta-analysis. JAMA Network Open. 2019http://dx.doi.org/10.1001/jamanetworkopen.2019.12458.

Macki M, Dabaja AA. Literature review of vaccine-related adverse events reported from HPV vaccination in randomized controlled trials. Basic and clinical andrology. 2016; 26: 16.

Macklin GR, Grassly NC, Sutter RW, Mach O, Bandyopadhyay AS, Edmunds WJ, et al. Vaccine schedules and the effect on humoral and intestinal immunity against poliovirus: a systematic review and network meta-analysis. The Lancet Infectious Diseases. 2019; 19: 1121-1128. http://dx.doi.org/10.1016/S1473-3099%2819%2930301-9.

Malisheni M, Khaiboullina SF, Rizvanov AA, Takah N, Murewanhema G, Bates M. Clinical efficacy, safety, and immunogenicity of a live attenuated tetravalent dengue vaccine (CYD-TDV) in children: A systematic review with meta-analysis. Frontiers in Immunology. 2017; 8: 863. http://dx.doi.org/10.3389/fimmu.2017.00863.

Mansour-Ghanaei R, Moradi-Lakeh M, Shakerian S, Karimi A, Esmaeeli S, Shokraneh F, et al. Acellular pertussis vaccine efficacy: An updated systematic review and meta -analysis. Medical journal of the Islamic Republic of Iran. 2016; 30: 451.

Marin M, Marti M, Kambhampati A, Jeram SM, Seward JF. Global varicella vaccine effectiveness: A meta-analysis. Pediatrics. 2016; 137: e20153741. http://dx.doi.org/10.1542/peds.2015-3741.

Marra F, Vadlamudi NK. Efficacy and safety of the pneumococcal conjugate-13 valent vaccine in adults. Aging and Disease. 2019; 10: 404-418. http://dx.doi.org/10.14336/AD.2018.0512.

McMillan M, Clarke M, Parrella A, Fell DB, Amirthalingam G, Marshall HS. Safety of tetanus, diphtheria, and pertussis vaccination during pregnancy a systematic review. Obstetrics and Gynecology. 2017; 129: 560-573. http://dx.doi.org/10.1097/AOG.0000000000001888.

Meggiolaro A, Migliara G, La Torre G. Association between Human Papilloma Virus (HPV) vaccination and risk of Multiple Sclerosis: A systematic review. Human Vaccines and Immunotherapeutics. 2018; 14: 1266-1274. http://dx.doi.org/10.1080/21645515.2017.1423155.

Mellone NG, Silva MT, Del Grossi Paglia M, Lopes LC, Barberato-Filho S, De Sa Del Fiol F, et al. Kawasaki disease and the use of the rotavirus vaccine in children: A systematic review and meta-analysis. Frontiers in Pharmacology. 2019; 10: 1075. http://dx.doi.org/10.3389/fphar.2019.01075.

Mertz D, Fadel SA, Lam P, Tran D, Srigley JA, Asner SA, et al. Herd effect from influenza vaccination in nonhealthcare settings: A systematic review of randomised controlled trials and observational studies. Eurosurveillance. 2016; 21http://dx.doi.org/10.2807/1560-7917.ES.2016.21.42.30378.

Milligan R, Paul M, Richardson M, Neuberger A. Vaccines for preventing typhoid fever. Cochrane Database of Systematic Reviews. 2018; 2018: CD001261. <http://dx.doi.org/10.1002/14651858.CD001261.pub4>.

Moa AM, Chughtai AA, Muscatello DJ, Turner RM, MacIntyre CR. Immunogenicity and safety of inactivated quadrivalent influenza vaccine in adults: A systematic review and meta-analysis of randomised controlled trials. Vaccine. 2016 Jul 29;34(35):4092-4102.doi: 10.1016/j.vaccine.2016.06.064.

Morgan E, Halliday SR, Campbell GR, Cardwell CR, Patterson CC. Vaccinations and childhood type 1 diabetes mellitus: a meta-analysis of observational studies. Diabetologia. 2016; 59: 237-243. http://dx.doi.org/10.1007/s00125-015-3800-8.

Morimoto N, Takeishi K. Change in the efficacy of influenza vaccination after repeated inoculation under antigenic mismatch: A systematic review and meta-analysis. Vaccine. 2018; 36: 949-957. https://dx.doi.org/10.1016/j.vaccine.2018.01.023.

Mouchet J, Salvo F, Raschi E, Poluzzi E, Antonazzo IC, De Ponti F, et al. Hepatitis B vaccination and the putative risk of central demyelinating diseases - A systematic review and meta-analysis. Vaccine. 2018; 36: 1548-1555. http://dx.doi.org/10.1016/j.vaccine.2018.02.036.

Mulley WR, Dendle C, Ling JEH, Knight SR. Does vaccination in solid-organ transplant recipients result in adverse immunologic sequelae? A systematic review and meta-analysis. Journal of Heart and Lung Transplantation. 2018; 37: 844-852. http://dx.doi.org/10.1016/j.healun.2018.03.001.

Mutsaerts EAML, Nunes MC, van Rijswijk MN, Klipstein-Grobusch K, Grobbee DE, Madhi SA. Safety and Immunogenicity of Measles Vaccination in HIV-Infected and HIV-Exposed Uninfected Children: A Systematic Review and Meta-Analysis. EClinicalMedicine. 2018; 1: 28-42. http://dx.doi.org/10.1016/j.eclinm.2018.06.002.

Ng TWY, Cowling BJ, Gao HZ, Thompson MG. Comparative Immunogenicity of Enhanced Seasonal Influenza Vaccines in Older Adults: A Systematic Review and Meta-analysis. The Journal of infectious diseases. 2019; 219: 1525-1535. http://dx.doi.org/10.1093/infdis/jiy720.

Ngocho JS, Magoma B, Olomi GA, Mahande MJ, Msuya SE, de Jonge MI, et al. Effectiveness of pneumococcal conjugate vaccines against invasive pneumococcal disease among children under five years of age in Africa: A systematic review. PLoS ONE. 2019; 14: e0212295. http://dx.doi.org/10.1371/journal.pone.0212295.

Nic Lochlainn LM, de Gier B, van der Maas N, van Binnendijk R, Strebel PM, Goodman T, et al. Effect of measles vaccination in infants younger than 9 months on the immune response to subsequent measles vaccine doses: a systematic review and meta-analysis. The Lancet Infectious Diseases. 2019; 19: 1246-1254. http://dx.doi.org/10.1016/S1473-3099%2819%2930396-2.

Norhayati MN, Ho JJ, Azman MY. Influenza vaccines for preventing acute otitis media in infants and children. Cochrane Database of Systematic Reviews. 2017; 2017: CD010089. http://dx.doi.org/10.1002/14651858.CD010089.pub3.

Pang Y, Zhao A, Cohen C, Kang W, Lu J, Wang G, et al. Current status of new tuberculosis vaccine in children. Human Vaccines and Immunotherapeutics. 2016; 12: 960-970. http://dx.doi.org/10.1080/21645515.2015.1120393.

Poudel S, Shehadeh F, Zacharioudakis IM, Tansarli GS, Zervou FN, Kalligeros M, et al. The effect of influenza vaccination on mortality and risk of hospitalization in patients with heart failure: A systematic review and meta-analysis. Open Forum Infectious Diseases. 2019; 6http://dx.doi.org/10.1093/ofid/ofz159.

Puges M, Biscay P, Barnetche T, Truchetet MF, Richez C, Seneschal J, et al. Immunogenicity and impact on disease activity of influenza and pneumococcal vaccines in systemic lupus erythematosus: A systematic literature review and meta-analysis. Rheumatology (United Kingdom). 2016; 55: 1664-1672. http://dx.doi.org/10.1093/rheumatology/kew211.

Restivo V, Costantino C, Bono S, Maniglia M, Marchese V, Ventura G, et al. Influenza vaccine effectiveness among high-risk groups: A systematic literature review and meta-analysis of case-control and cohort studies. Human Vaccines and Immunotherapeutics. 2018; 14: 724-735. http://dx.doi.org/10.1080/21645515.2017.1321722.

Rodrigues BS, David C, Costa J, Ferreira JJ, Pinto FJ, Caldeira D. Influenza vaccination in patients with heart failure: A systematic review and meta-analysis of observational studies. Heart. 2019http://dx.doi.org/10.1136/heartjnl-2019-315193.

Rodrigues IC, De Da Silva RCMA, De Felicio HCC, Da Silva RF. New immunization schedule effectiveness against hepatitis B in liver transplantation patients. Arquivos de Gastroenterologia. 2019; 56: 440-446. http://dx.doi.org/10.1590/s0004-2803.201900000-77.

Rondy M, El Omeiri N, Thompson MG, Leveque A, Moren A, Sullivan SG. Effectiveness of influenza vaccines in preventing severe influenza illness among adults: A systematic review and meta-analysis of test-negative design case-control studies. Journal of Infection. 2017; 75: 381-394. http://dx.doi.org/10.1016/j.jinf.2017.09.010.

Rosa BR, Cunha AJLAD, Medronho RDA. Efficacy, immunogenicity and safety of a recombinant tetravalent dengue vaccine (CYD-TDV) in children aged 2-17 years: Systematic review and meta-analysis. BMJ Open. 2019; 9: e019368. http://dx.doi.org/10.1136/bmjopen-2017-019368.

Schwartz KL, Kwong JC, Deeks SL, Campitelli MA, Jamieson FB, Marchand-Austin A, et al. Effectiveness of pertussis vaccination and duration of immunity. CMAJ. 2016; 188: E399-E406. http://dx.doi.org/10.1503/cmaj.160193.

Schwerdtle P, Onekon CK, Recoche K. A Quantitative Systematic Review and Meta-Analysis of the Effectiveness of Oral Cholera Vaccine as a Reactive Measure in Cholera Outbreaks. Prehospital and disaster medicine. 2018; 33: 2-6. http://dx.doi.org/10.1017/S1049023X17007166.

Senderovich H, Grewal J, Mujtaba M. Herpes zoster vaccination efficacy in the long-term care facility population: a qualitative systematic review. Current Medical Research and Opinion. 2019; 35: 1451-1462. http://dx.doi.org/10.1080/03007995.2019.1600482.

Simancas-Racines D, Franco JV, Guerra CV, Felix ML, Hidalgo R, Martinez-Zapata MJ. Vaccines for the common cold. Cochrane Database of Systematic Reviews. 2017; 2017: CD002190. http://dx.doi.org/10.1002/14651858.CD002190.pub5.

Soares-Weiser K, Bergman H, Henschke N, Pitan F, Cunliffe N. Vaccines for preventing rotavirus diarrhoea: Vaccines in use. Cochrane Database of Systematic Reviews. 2019; 2019: CD008521. http://dx.doi.org/10.1002/14651858.CD008521.pub4.

Stassijns J, Bollaerts K, Baay M, Verstraeten T. A systematic review and meta-analysis on the safety of newly adjuvanted vaccines among children. Vaccine. 2016; 34: 714-722. http://dx.doi.org/10.1016/j.vaccine.2015.12.024.

Tang G, Yin W, Tan L, Wu S, Cao Y, Fu X, et al. Immunogenicity of sequential inactivated and oral poliovirus vaccines (OPV) versus inactivated poliovirus vaccine (IPV) alone in healthy infants: A systematic review and meta-analysis. Human Vaccines and Immunotherapeutics. 2018; 14: 2636-2643. http://dx.doi.org/10.1080/21645515.2018.1489188.

Tejada RA, Vargas KG, Benites-Zapata V, Mezones-Holguin E, Bolanos-Diaz R, Hernandez AV. Human papillomavirus vaccine efficacy in the prevention of anogenital warts: systematic review and meta-analysis. Salud publica de Mexico. 2017; 59: 84-94. http://dx.doi.org/10.21149/7824.

Tricco AC, Zarin W, Cardoso R, Veroniki AA, Khan PA, Nincic V, et al. Efficacy, effectiveness, and safety of herpes zoster vaccines in adults aged 50 and older: Systematic review and network meta-analysis. BMJ (Online). 2018; 363: k4029. http://dx.doi.org/10.1136/bmj.k4029.

Tsivgoulis G, Katsanos AH, Zand R, Ishfaq MF, Malik MT, Karapanayiotides T, et al. The association of adult vaccination with the risk of cerebrovascular ischemia: A systematic review and meta-analysis. Journal of the Neurological Sciences. 2018; 386: 12-18. http://dx.doi.org/10.1016/j.jns.2018.01.007.

Vadlamudi NK, Parhar K, Altre Malana KL, Kang A, Marra F. Immunogenicity and safety of the 13-valent pneumococcal conjugate vaccine compared to 23-valent pneumococcal polysaccharide in immunocompetent adults: A systematic review and meta-analysis. Vaccine. 2019; 37: 1021-1029. http://dx.doi.org/10.1016/j.vaccine.2019.01.014.

Vardanjani HM, Borna H, Ahmadi A. Effectiveness of pneumococcal conjugate vaccination against invasive pneumococcal disease among children with and those without HIV infection: A systematic review and meta-analysis. BMC Infectious Diseases. 2019; 19: 685. http://dx.doi.org/10.1186/s12879-019-4325-4.

Vasileiou E, Sheikh A, Butler C, El Ferkh K, Von Wissmann B, McMenamin J, et al. Efectiveness of influenza vaccines in Asthma: A systematic review and meta-analysis. Clinical Infectious Diseases. 2017; 65: 1388-1395. http://dx.doi.org/10.1093/cid/cix524.

Vasileiou E, Simpson C, Sheikh A, Butler C. Seasonal influenza vaccine effectiveness in people with asthma: A systematic review. European Respiratory Journal. 2016; 48http://dx.doi.org/10.1183/13993003.congress-2016.PA4205.

Vinkenes E, Nielsen MA, Blaakaer J. Is there evidence for efficacy of human papillomavirus vaccination in solid organ transplant recipients? European Journal of Obstetrics and Gynecology and Reproductive Biology: X. 2019; 4: 100015. http://dx.doi.org/10.1016/j.eurox.2019.100015.

Voysey M, Sadarangani M, Clutterbuck E, Bolgiano B, Pollard AJ. The impact of administration of conjugate vaccines containing cross reacting material on Haemophilus influenzae type b antibody responses in infants: A systematic review and meta-analysis of randomised controlled trials. Vaccine. 2016; 34: 3986-3992. http://dx.doi.org/10.1016/j.vaccine.2016.06.038.

Whitford K, Liu B, Micallef J, Yin JK, Macartney K, Damme PV, et al. Long-term impact of infant immunization on hepatitis B prevalence: A systematic review and meta-analysis. Bulletin of the World Health Organization. 2018; 96: 484-497. http://dx.doi.org/10.2471/BLT.17.205153.

Xu J, Liu S, Liu Q, Rong R, Tang W, Wang Q, et al. The effectiveness and safety of pertussis booster vaccination for adolescents and adults: A systematic review and meta-analysis. Medicine. 2019; 98: e15281. http://dx.doi.org/10.1097/MD.0000000000015281.

Xu L, Selk A, Garland SM, Bogliatto F, Kyrgiou M, Weyers S, et al. Prophylactic vaccination against human papillomaviruses to prevent vulval and vaginal cancer and their precursors. Expert Review of Vaccines. 2019; 18: 1157-1166. http://dx.doi.org/10.1080/14760584.2019.1692658.

Yakely AE, Avni-Singer L, Oliveira CR, Niccolai LM. Human Papillomavirus Vaccination and Anogenital Warts: A Systematic Review of Impact and Effectiveness in the United States. Sexually Transmitted Diseases. 2019; 46: 213-220. http://dx.doi.org/10.1097/OLQ.0000000000000948.

Yin M, Xu X, Liang Y, Ni J. Effectiveness, immunogenicity and safety of one vs. two-dose varicella vaccination:a meta-analysis. Expert Review of Vaccines. 2018; 17: 351-362. http://dx.doi.org/10.1080/14760584.2018.1433999.

Zhan Y, Liu X, Feng Y, Wu S, Jiang Y. Safety and efficacy of human papillomavirus vaccination for people living with HIV: A systematic review and meta-analysis. International Journal of STD and AIDS. 2019; 30: 1105-1115. http://dx.doi.org/10.1177/0956462419852224.

Zhang C, Wang X, Liu D, Zhang L, Sun X. A systematic review and meta-analysis of fetal outcomes following the administration of influenza A/H1N1 vaccination during pregnancy. International Journal of Gynecology and Obstetrics. 2018; 141: 141-150. http://dx.doi.org/10.1002/ijgo.12394.

Zheng D, Gao F, Zhao C, Ding Y, Cao Y, Yang T, et al. Comparative effectiveness of H7N9 vaccines in healthy individuals. Human Vaccines and Immunotherapeutics. 2019; 15: 80-90. http://dx.doi.org/10.1080/21645515.2018.1515454.

Zimmermann P, Finn A, Curtis N. Does BCG vaccination protect against nontuberculous mycobacterial infection? A systematic review and meta-analysis. Journal of Infectious Diseases. 2018; 218: 679-687. http://dx.doi.org/10.1093/infdis/jiy207.
