# Supplementary material for: Impact of industry sponsorship on the quality of systematic reviews of vaccines: a cross-sectional analysis of studies published from 2016 to 2019
Source: Syst Rev. 2022 Aug 22;11:174. doi: 10.1186/s13643-022-02051-x (PMC9395849; doi:10.1186/s13643-022-02051-x)
Supplement: Supplementary file 4 — Additional file 4. Characteristics of included SRs. [file 13643_2022_2051_MOESM4_ESM.docx]

Appendix 4: characteristics of included SRs

Industry funded SRs

| **SR** | **country** | **journal (impact factor)** | **number of studies** | **types of studies included** | **number of data-bases** | **intervention** | **outcomes** | **quality appraisal tool** | **meta-analysis** | **Hetero-geneity assessed** | **publica-tion bias assessed** | **conflict of interest reported** | **financial support** |
| --- | --- | --- | --- | --- | --- | --- | --- | --- | --- | --- | --- | --- | --- |
| **Bakker et al. 2016** | Belgium | Expert Review of Vaccines (4.362) | 47 | RCTs and NRSI | 4 | Twinrix, Twinrix PediatricTM and AmbirixTM against HBV and HAV | effectiveness/efficacy, immunogenicity, safety | / | no | no | no | yes | GlaxoSmithKline Biologicals S.A. |
| **Caspard et al. 2016** | USA | Human Vaccines & Immunotherapeutics (2.619) | 4 | RCTs | 2 | live attenuated influenza vaccine (LAIV) | effectiveness/efficacy | / | yes | no | no | yes | Med-Immune, AstraZeneca |
| **Schiffner- Rohe et al. 2016** | Germany | PLoS One (2.740) | 4 | RCTs | 3 | PPV23 | effectiveness/efficacy | Cochrane Risk of Bias tool | yes | yes | no | yes | Pfizer Deutschland GmbH |
| **Bekkat-Berkani et al. 2017** | Belgium | BMC Pulm Med (2.813) | 17 | RCTs and NRSI | 6 | Seasonal influenza vaccination | immunogenicity, safety, effectiveness/ efficacy | Scottish Inter-collegiate Guidelines Network | no | no | no | yes | GlaxoSmithKline Biologicals SA |
| **Cafiero-Fonseca et al. 2017** | USA | PLoS One (2.740) | 150 | RCTs and NRSI | 2 | Vaccination with PCV13 or PPV23 | effectiveness/efficacy | / | no | no | no | yes | Pfizer Inc. |
| **Caspard et al. 2017** | USA | Open Forum Infectious Diseases (3.656) | 24 | NRSI | 1 | Live-Attenuated Influenza Vaccine (LAIV) | effectiveness/ efficacy | / | yes | yes | no | yes | Astra-Zeneca |
| **Lansbury et al. 2017** | UK | Vaccine (3.143) | 38 | NRSI | 8 | pandemic influenza A(H1N1) vaccines | effectiveness/efficacy | Cochrane Collabora-tion tool, Newcastle-Ottawa scale | yes | yes | yes | yes | GlaxoSmithKline Biologicals SA |
| **Sousa et al. 2017** | Portugal | ACTA REUMATOL PORT (1.183) | 28 | RCTs and NRSI | 2 | various vaccines | effectiveness/efficacy, safety | Cochrane Collabora-tion tool | no | no | no | no | Sanofi-Pasteur, MSD |
| **Tin Tin Htar et al. 2017** | France | PLoS One (2.740) | 33 | NRSI | 3 | PPV23 or PCV13 | effectivness/ efficacy | Newcastle-Ottawa scale | yes | yes | yes | yes | Pfizer, Paris |
| **Van Den Ende et al. (children) 2017** | Belgium | Expert Review of Vaccines (4.362) | 60 | RCTs and NRSI | 4 | GSK’s recombinant hepatitis B vaccine | effectiveness/ efficacy, immuno-genicity and safety | / | no | no | no | yes | GSK Biologicals S.A, Belgium |
| **Van Den Ende et al. (adults) 2017** | Belgium | Expert Review of Vaccines (4.362) | 64 | RCTs and NRSI | 4 | GSK’s recombinant hepatitis B vaccine | effectiveness/ efficacy, immuno-genicity and safety | / | no | no | no | yes | GSK Biologicals S.A, Belgium |
| **Chit et al. 2018** | Canada | PLoS One (2.740) | 7 | NRSI | 4 | acellular pertussis vaccinantion | effectiveness/ efficacy | modified "Black and Downs" checklist | yes | yes | yes | yes | Sanofi Pasteur und Mitacs |
| **Dos Santos et al. 2018** | Belgium | Human Vaccines & Immunotherapeutics (2.619) | 15 | RCTs and NRSI | 5 | influenza vaccination | immuno-genicity, safety, effectiveness/ efficacy | Scottish Intercolle-giate Guidelines Network (SIGN) | no | no | no | yes | GlaxoSmithKline Biologicals (GSK) |
| **Lee et al. 2018** | Canada | Expert Review of Vaccines (4.362) | 8 | RCTs and NRSI | 3 | HD-IIV3 influenza vaccination | effectiveness/ efficacy | modified "Black and Downs" checklist | yes | yes | yes | yes | Sanofi Pasteur |
| **Patterson et al. 2018** | South Africa | Vaccine (3.143) | 47 | RCTs and NRSI | 10 | wP vaccination against pertussis | safety | CASP checklist, Cochrane Risk of bias tool | yes | yes | no | no | Sanofi Pasteur |
| **Steben et al. 2018** | Canada | Journal of Obstetrics and Gynaecology Canada (-) | 7 | NRSI | 1 | quadrivalent HPV vaccination | effectiveness/ efficacy | / | no | no | no | yes | Merck Canada |
| **Willame et al. 2018** | Belgium | Open Forum Infectious Diseases (3.656) | 32 | NRSI | 4 | Rotarix against rotavirus | effectiveness/ efficacy | Coordination of Cancer Clinical Practice Guidelines | yes | yes | yes | yes | GlaxoSmithKline Biologicals (GSK) |
| **Bravo et al. 2019** | France | Expert Review of Vaccines (4.362) | 11 | RCTs and NRSI | 2 | Inactivated HAV pediatric vaccine (Avaxim® 80U Pediatric, Sanofi Pasteur) | Immunogenicity and safety | / | no | no | no | yes | Sanofi Pasteur |
| **D'Heilly et al. 2019** | France | Infectious Diseases and Therapy (4.307) | 27 | RCTs and NRSI | 6 | Pertussis vaccines | safety | / | no | no | no | yes | Sanofi Pasteur |
| **Dolhain et al. 2019** | Belgium | Expert Review of Vaccines (4.362) | 6 | RCTs | 1 | Hexavalent diphtheria, tetanus, acellular pertussis, hepatitis B virus, inactivated poliomyelitis and Haemophilus influenzae type b conjugate vaccine | Immunogenicity and safety | / | no | no | no | yes | GlaxoSmithKline Biologicals SA |
| **McGirr et al. 2019** | Canada | Vaccine (3.143) | 21 | RCTs | 4 | Adjuvanted recombinant zoster vaccine (RZV) and zoster vaccine live (ZVL) | effectiveness/efficacy and safety | Cochrane Risk of Bias Tool | yes | yes | no | yes | GlaxoSmithKline Biologicals SA |
| **McLaughlin et al. 2019** | USA | Vaccine (3.143) | 3 | RCTs and NRSI | 1 | Pneumococcal conjugate vaccine (PCV13) | effectiveness/ efficacy | / | yes | yes | no | yes | Pfizer Inc |
| **Nieto Guevara et al. 2019** | Panama | Expert Review of Vaccines (4.362) | 7 | RCTs and NRSI | 4 | Pneumococcal non-typeable Haemophilus influenzae protein D-conjugate vaccine (PHiD-CV) and 13-valent PCV (PCV13) | effectiveness/ efficacy, safety, and immunogenicity | Cochrane Risk of Bias Tool, STROBE-Checklist | no | no | no | yes | GlaxoSmithKline Biologicals SA |
| **Samson et al. 2019** | USA | Expert Review of Vaccines (4.362) | 25 | RCTs | 1 | High-dose trivalent, inactivated, split-virus influenza vaccine (IIV3-HD) | Immunogenicity | Jadad scale | yes | yes | no | yes | Sanofi Pasteur |
| **Sings et al. 2019** | USA | Clinical Infectious Diseases (8.313) | 8 | NRSI | 5 | Pneumococcal conjugate vaccine (PCV13) | effectiveness/ efficacy | Newcastle-Ottawa Scale | yes | yes | no | yes | Pfizer Inc |
| **Switzer et al. 2019** | Canada | Infectious Diseases and Therapy (4.307) | 43 | RCTs and NRSI | 6 | Maternal pertussis vaccination during pregnancy (tetanus toxoid, reduced-dose diphtheria toxoid, and reduced-dose acellular pertussis [Tdap] vaccine and Tdap-inactivated polio vaccine [IPV]) | effectiveness/ efficacy and Immunogenicity | / | no | no | no | yes | Sanofi Pasteur |
| **Tin Tin Htar et al. 2019** | France | Expert Review of Vaccines (4.362) | 99 | RCTs and NRSI | 1 | PCV7, PCV10, and PCV13 | effectiveness/efficacy | / | no | no | no | yes | Pfizer Inc |

DT diphtheria; HAV Hepatitis A; GSK GlaxoSmithKline Biologicals; HBV Hepatitis B virus; HD-IIV3 High-dose inactivated trivalent influenza vaccine; HPV Human Papilloma-virus; IPV inactivated polio vaccine; LAIV live attenuated influenza vaccine ; MMR measels/ mumps/ rubella; NRSI non-ramdomized studies; PCV 7 7 valent pneumococcal conjugate vaccine; PCV 10 10 valent pneumococcal conjugate vaccine; PCV 13 13 valent pneumococcal conjugate vaccine PHiD-CV PPV23 23-Valent Pneumococcal Polysaccharide Vaccine; PPV24 24-Valent Pneumococcal Polysaccharide Vaccine; RCTs randomized controlled trials; RZV recombinant zoster vaccine; STROBE Strengthening the Reporting of Observational Studies in Epidemiology; Tdap tetanus toxoid, reduced-dose diphtheria toxoid, and reduced-dose acellular pertussis; wP whole-cell pertussi ; ZVL zoster vaccine live

Non-industry funded SRs

| **SR** | **country** | **journal (impact factor)** | **number of studies** | **types of studies included** | **number of data-bases** | **intervention** | **outcomes** | **quality appraisal tool** | **meta-analysis** | **Hetero-geneity assessed** | **publica-tion bias assessed** | **conflict of interest reported** | | **financial support** |
| --- | --- | --- | --- | --- | --- | --- | --- | --- | --- | --- | --- | --- | --- | --- |
| **De Oliveira et al. 2016** | United Kingdom | PLOS ONE (2.740) | 22 | NRSI | 5 | PCV-10 or PCV-13 | effectiveness/ efficacy, safety, and immunogenicity | NOS, NIH checklist, modified vision of Ramsey et al. criteria | no | no | no | | no | Pan American Health Organization and the Sabin Vaccine Institute |
| **Ewald et al. 2016** | Switzerland | Dtsch Arztebl Int (4.796) | 21 | RCTs | 2 | PCVs alone or in combination with other non-pneumococcal vaccines | effectiveness/efficacy | Cochrane Risk of Bias Tool | yes | yes | yes | | no | none |
| **Liao et al. 2016** | China | PLOS ONE (2.740) | 18 | NRSI | 7 | Influenza Vaccination | safety, immunogenicity | / | yes | yes | yes | | no | Science Foundation of Dongguan and special fund for discipline construction of colleges and universities in Guangdong Province |
| **Nunes et al. 2016** | South Africa | AM J PERINAT (1.474) | 23 | RCTs and NRSI | 1 | Influenza Vaccination, A/H1N1pdm09 influenza vaccine | safety | / | yes | yes | yes | | no | Bill & Melinda Gates Foundation, the South African Research Chairs Initiative of the Department of Science and Technology and National Research Foundation in Vaccine Preventable Diseases; and the Medical Research Council: Respiratory and Meningeal Pathogens Research Unit |
| **Remschmidt et al. 2016** | Germany | BMC Infect Dis (2.688) | 14 | RCTs and NRSI | 3 | PPSV-23 | effectiveness/ efficacy | Cochrane risk of bias tool | no | no | no | | no | none |
| **Santos et al. 2016** | Brazil | Infect Dis Poverty (3.067) | 9 | NRSI | 3 | rotavirus vaccines | effectiveness/ efficacy | / | yes | yes | yes | | no | public funding |
| **Azami et al. 2017** | Iran | Hepatitis Monthly (0.705) | 19 | NRSI | 18 | Hepatitis B Vaccination | effectiveness/ efficacy | STROBE | Yes | Yes | Yes | | no | sponsered by Ilam University of Medical Sciences |
| **D'Addario et al. 2017** | Switzerland | Vaccine (3.143) | 7 | RCTs and NRSI | 3 | bivalent vaccine against HPV types 16 and 18 or quadrivalent vaccine against HPV types 6, 11, 16 and 18 | immunogenicity | / | Yes | Yes | No | | no | grants from the World Health Organization and the Swiss National Science Foundation |
| **Falkenhorst, G., et al. 2017** | Germany | PLOS ONE (2.740) | 17 | RCTs and NRSI | 3 | PPV23 | effectiveness/ efficacy | Cochrane risk of bias tool, NOS | Yes | No | No | | no | funded by the authors’ respective institutional budgets |
| **Godoi et al. 2017** | Sweden | Journal of comparative effectiveness research (1.468) | 10 | RCTs | 3 | CYD-TDV Dengue Vaccine | effectiveness/ efficacy, immunogenicity and safety | Cochrane Risk of Bias Tool | Yes | Yes | No | | no | financial support from CAPES |
| **Kassim, P., et al. 2017** | Australia | Vaccine (3.143) | 11 | NRSI | 2 | rotavirus vaccination | safety | NOS | Yes | Yes | Yes | | no | none |
| **Lee, K.R., et al. 2017** | Republic of Korea | Neuroepidemiology (2.186) | 11 | NRSI | 5 | Influenza Vaccination | effectiveness/ efficacy | NOS | Yes | Yes | Yes | | no | funded by the Ministry of Health and Welfare thorough the Korea Health Industry Development Institute |
| **Ogawa, Y., et al. 2017** | Japan | journal of pharmaceutical health care and sciences (-) | 24 | RCTs | 3 | three types of HPV vaccines | safety | Cochrane Risk of Bias Tool | Yes | Yes | No | | no | none |
| **Wilkinson, K., et al. 2017** | Canada | Vaccine (3.143) | 8 | RCTs | 5 | High-dose inactivated TIV administered intramuscularly | immunogenicity and seroprotection | Cochrane Risk of Bias Tool | Yes | Yes | No | | no | none |
| **Dzanibe et al. 2018** | South Africa | Expert Review of Vaccines (4.362) | 25 | RCTs and NRSI | 5 | CPS-based vaccine against GBS | safety, immunogenicity | / | no | no | no | | yes | none |
| **Genovese et al. 2018** | Italy | Journal of Preventive Medicine and Hygiene (-) | 6 | RCTs | 4 | HPV vaccine | safety | Jadad scale | yes | yes | no | | no | none |
| **Harder et al. 2018** | Germany | BMC Medicine (6.782) | 7 | RCTs and NRSI | 3 | HPV vaccine | effectiveness/ efficacy, safety | Cochrane Risk of Bias Tool, ROBINS-I | no | no | no | | no | none |
| **Imai et al. 2018** | Australia | PLoS One (2.740) | 13 | RCTs and NRSI | 3 | seasonal Influenza vaccine | effectiveness/ efficacy | Cochrane Risk of Bias Tool; NOS | yes | yes | no | | no | Australian National Health and Medical Research Council for Centre of Research Excellence Grant; Australian Centre for Health Services Innovation |
| **Mouchet et al. 2018** | France | Pharmacological Research (5.893) | 25 | RCTs and NRSI | 6 | HPV vaccine | safety | NOS | yes | yes | no | | no | University Bordeaux Frankreich und INSERM (National Institute of Medical Research) |
| **Xu et al. 2018** | China | Human Vaccines & Immunotherapeutics (2.619) | 3 | RCTs | 5 | SA4Ag and SA3Ag against staphylococcus | safety | Cochrane Risk of Bias Tool | yes | yes | yes | | no | National Research Foundation for Health and Family Planning Commission of China und Key Research and Development Project of Zhejiang Provincial Science and Technology Department |
| **Adetokunboh et al. 2019** | South Africa | Human Vaccines and Immunotherapeutics (2.619) | 14 | RCTs and NRSI | 6 | BCG, HBV, oral polio vaccine, inactivated polio vaccine, diphtheria-tetanus- pertussis containing vaccines, Hib, PCV, RV, yellow fever vaccine and measles containing vaccines | effectiveness/ efficacy | Cochrane risk of bias tool | Yes | Yes | No | | no | none |
| **Caldeira et al. 2019** | Portugal | Expert Review of Vaccines (4.362) | 5 | NRSI | 9 | Influenza vaccination | Safety | / | Yes | Yes | No | | yes | none |
| **GarciaPerdomo et al. 2019** | Colombia | Epidemiology and infection (2.152) | 29 | RCTs | 4 | HPV-vaccines (bivalent vaccine/tetravalent/nanovalent) | effectiveness/ efficacy | Cochrane risk of bias tool | Yes | Yes | No | | no | none |
| **Ji et al. 2019** | China | Frontiers in Immunology (5.085) | 7 | RCTs | 5 | Tuberculosis vaccination (M72/AS01E Candidate Vaccine) | Immunogenicity and Safety | Cochrane risk of bias tool | Yes | Yes | Yes | | no | none |
| **Lindsey et al. 2019** | United Kindgom | The Lancet Infectious Diseases (24.446) | 23 | RCTs and NRSI | 4 | Influenza vaccination | effectiveness/ efficacy, and immunogenicity | GRADE | No | Yes | No | | no | none |
| **Mehtani et al. 2019** | USA | American Journal of Epidemiology (4.526) | 12 | RCTs and NRSI | 3 | Measles vaccination | Immunogenicity and safety | NOS | No | Yes | No | | no | none |
| **Nic Lochlainn et al. 2019** | Nether-lands | The Lancet Infectious Diseases (24.446) | 56 | RCTs and NRSI | 7 | Measles vaccination | effectiveness/ efficacy, immunogenicity, and safety | GRADE | Yes | Yes | No | | no | WHO |
| **Sinzinger et al. 2019** | Germany | Human Vaccines and Immunotherapeutics (2.619) | 5 | NRSI | 2 | MMR vaccine | effectiveness/ efficacy | ROBINS-I | Yes | Yes | No | | no | internal resources of the second author’s (RvK) department |
| **Tan et al. 2019** | China | BMC Pregnancy and Childbirth (2.413) | 7 | NRSI | 3 | HPV vaccination | Safety | NOS | Yes | Yes | No | | no | public funding |
| **Wang et al. 2019** | China | Human Vaccines and Immunotherapeutics (2.619) | 8 | RCTs and NRSI | 6 | HPV vaccination | Safety | NOS, Jadad scale | Yes | No | Yes | | no | public funding |

BCG Bacillus Calmette-Guérin; CAPES Coordenação de Aperfeiçoamento de Pessoal de Nível Superior; CPS capsular polysaccharides DT diphtheria; CYD- chimeric yellow fever-dengue tetravalent dengue vaccine; NRSI non-ramdomized studies; GBS group B Streptococcus GRADE grading of recommendation, assessment, development and evaluation ;HAV Hepatitis A virus; Hib Haemophilus influenzae Typ b; HBV Hepatitis B virus; HD-IIV3 High-dose inactivated trivalent influenza vaccine; HPV Human Papilloma-virus, MMR measels/ mumps/ rubella; NOS Newcastle-Ottawa Scale; PCV pneumococcal conjugate vaccine; PCV 10 10 valent pneumococcal conjugate vaccine00; PCV 13 13 valent pneumococcal conjugate vaccine; PPV23 23-Valent Pneumococcal Polysaccharide Vaccine; PPV24 24-Valent Pneumococcal Polysaccharide Vaccine; RCTs randomized controlled trials; ROBINS I risk of bias in non-randomized studies – of interventions; RV rotavirus; SA3Ag Staphylococcus aureus 3-antigen vaccine; SA4Ag Staphylococcus aureus 4-antigen vaccine; STROBE Strengthening The Reporting of Observational Studies in Epidemiology; WHO World Health Organization
